# Supplementary material for: Targeting Gi/o-coupled GPCRs to inhibit nociceptors: Insights from the serotonin receptor Htr1b and triptans
Source: Cell Rep Med. 2026 Jul 30;7(8):102949. doi: 10.1016/j.xcrm.2026.102949 (PMC13522807; doi:10.1016/j.xcrm.2026.102949)
Supplement: Supplementary file 2 [file mmc2.pdf]

Cell Reports
Medicine

Article

Targeting $G_{i/o}$-coupled GPCRs to inhibit nociceptors: Insights from the serotonin receptor Htr1b and triptans

Graphical abstract

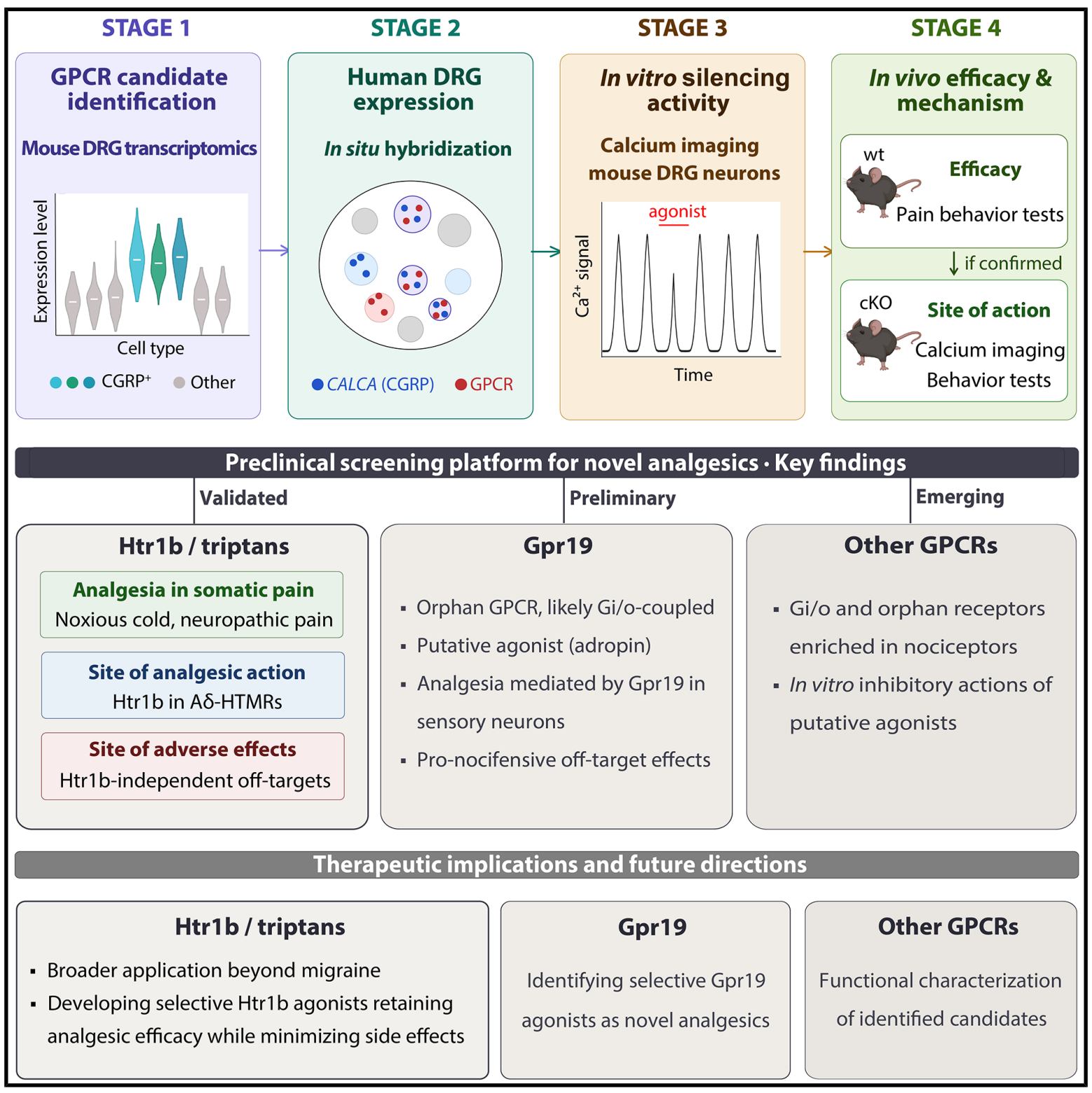

Highlights

- $G_{i/o}$-coupled GPCRs enriched in CGRP$^+$ mouse and human DRG neurons are identified
- Agonists of $G_{i/o}$-coupled GPCRs attenuate mouse nociceptor excitability *in vitro*
- Triptans reduce specific pain-related behaviors through Htr1b in DRG neurons
- The orphan GPCR Gpr19 emerges as a candidate analgesic target

Authors

Jing Peng, Brianna T. Sanchez, Anda M. Chirila, ..., Clifford J. Woolf, Nikhil Sharma, David D. Ginty

Correspondence

david_ginty@hms.harvard.edu

In brief

Peng et al. develop a preclinical screening platform that identifies $G_{i/o}$-coupled and orphan GPCRs enriched in CGRP$^+$ mouse and human DRG neurons. Using Htr1b and triptans as proof of principle, they show that activating $G_{i/o}$-coupled GPCRs attenuates nociceptor excitability and reduces specific pain-related behaviors in mice.

Peng et al., 2026, Cell Reports Medicine 7, 102949
August 18, 2026 © 2026 The Author(s). Published by Elsevier Inc.
https://doi.org/10.1016/j.xcrm.2026.102949

CellPress

Cell Reports Medicine

CellPress OPEN ACCESS

Article

Targeting $G_{i/o}$-coupled GPCRs to inhibit nociceptors: Insights from the serotonin receptor Htr1b and triptans

Jing Peng,[1,2] Brianna T. Sanchez,[1,2] Anda M. Chirila,[1,2,4] Xiangsunze Zeng,[1,3] Michelle M. DeLisle,[1,2] Lijun Qi,[1,2] Jia Yin Xiao,[1,2] Karina Lezgiyeva,[1,2] Sarah A. Low,[1,2,5] Clifford J. Woolf,[1,3] Nikhil Sharma,[1,2,6] and David D. Ginty[1,2,7,*]

[1]Department of Neurobiology, Harvard Medical School, Boston, MA 02115, USA
[2]Howard Hughes Medical Institute, Harvard Medical School, Boston, MA 02115, USA
[3]F.M. Kirby Neurobiology Center, Boston Children's Hospital, Boston, MA 02115, USA
[4]Department of Neuroscience, Brown University, Providence, RI 02912, USA
[5]Department of Anesthesiology, Critical Care and Pain Medicine, Boston Children's Hospital, Boston, MA 02115, USA
[6]Department of Biochemistry & Molecular Biophysics, Columbia University, New York, NY 10032, USA
[7]Lead contact
*Correspondence: david_ginty@hms.harvard.edu
https://doi.org/10.1016/j.xcrm.2026.102949

SUMMARY

Pain perception is initiated upon activation of nociceptors of the dorsal root ganglia (DRG) and trigeminal ganglia. We identify G-protein-coupled receptors (GPCRs) expressed in $CGRP^+$ mouse and human DRG neurons and find that agonists of several identified $G_{i/o}$-coupled and orphan GPCRs attenuate neuronal excitability. Experiments focusing on the $G_{i/o}$-coupled serotonin receptor Htr1b, which is expressed in mouse and human $CGRP^+$ DRG neurons, reveal that Htr1b/1d agonists, the triptans sumatriptan and zolmitriptan, attenuate $CGRP^+$ neuron excitability *in vitro* and exhibit analgesia across several pain models, including neuropathic pain. Conditional genetic deletion experiments show that triptan-induced analgesia is mediated by Htr1b expressed in A-fiber mechanonociceptors. Also, triptan-associated adverse effects are partially mediated by Htr1b-independent targets. Further testing identifies the GPCR Gpr19 as an additional promising target for treating pain. These findings establish a preclinical screening platform for identifying analgesic candidates and reveal nociceptor GPCRs that may be targeted to treat pain.

INTRODUCTION

Detection of noxious stimuli and the perception of pain are essential for avoiding injury. However, pain, defined as "an unpleasant sensory and emotional experience associated with, or resembling that associated with, actual or potential tissue damage" by the International Association for the Study of Pain,[1] can be debilitating and reduces quality of life. Current treatments often lack efficacy and carry significant adverse effects. Some treatments, notably opioids, although effective, contribute to the addiction epidemic due to their abuse liability. There is therefore an urgent need for understanding the underlying mechanisms of pain and developing treatments.

The perception of pain is initiated by activation of a specific subset of primary sensory neurons called nociceptors whose cell bodies reside within the trigeminal or dorsal root ganglia (DRG).[2] These sensory neurons express many G-protein-coupled receptors (GPCRs),[3–5] a large family of transmembrane proteins that respond to diverse extracellular stimuli and initiate intracellular signaling cascades.[6] Activation of GPCRs in nociceptors can increase or decrease neuronal excitability, augmenting or diminishing pain transduction.[7–10] The $G_{i/o}$-coupled subclass, which includes opioid receptors and metabotropic GABA receptors, inhibits adenylate cyclase and stimulates G-protein-activated inwardly rectifying K^+ (GIRK) channels, thereby reducing neuronal excitability. Activating $G_{i/o}$-coupled receptors in nociceptors may therefore attenuate pain.[11,12] Indeed, as the mainstream therapy against severe pain, morphine and other opioids target $G_{i/o}$-coupled opioid receptors.[13] Agonists of the $G_{i/o}$-coupled serotonin receptors Htr1b/1d, the triptans, are the first-line migraine treatments.[14] The $G_{i/o}$-coupled α2-adrenergic receptor agonists, including clonidine and dexmedetomidine, are used clinically as adjuvants in local anesthesia.[15,16] Despite their broad clinical use, the cellular site of action of these medications is unclear.

Over the last decade, major progress has been made toward understanding the physiological and functional properties of somatosensory neuron subtypes, and recent transcriptomic approaches have revealed the gene expression profiles of these physiologically defined neuronal subtypes,[4,5,17–19] enabling generation of genetic tools to manipulate select sensory neuron subtypes including nociceptors.[5,18] These findings may be exploited to test the hypothesis that activation of $G_{i/o}$-coupled GPCRs selectively expressed in nociceptors reduces pain without affecting touch or proprioception.

Here, we combined somatosensory neuron transcriptomics with *in vitro* calcium imaging to identify $G_{i/o}$-coupled GPCRs

© 2026 The Author(s). Published by Elsevier Inc.
This is an open access article under the CC BY license (http://creativecommons.org/licenses/by/4.0/).

expressed in mouse nociceptors and whose activation attenuates nociceptor excitability. Using Htr1b and triptans as a proof of principle, we employed mouse genetics, electrophysiology, and a range of animal behavioral paradigms to show that activating this $G_{i/o}$-coupled GPCR inhibits nociceptor excitability, reduces transmitter release from nociceptor terminals, and attenuates pain behaviors under normal and neuropathic pain conditions. Conditional genetic deletion experiments revealed that the analgesic effects of triptans are mediated by Htr1b in sensory neurons, while adverse effects are largely attributed to other targets. We also identified an orphan GPCR, Gpr19, as a $G_{i/o}$-coupled receptor and candidate analgesic target. Together, we propose revisiting the therapeutic utility of triptans beyond migraine, developing more selective Htr1b agonists that retain analgesic efficacy with minimized adverse effects, and future identification and testing of Gpr19 agonists and other identified GPCRs as potential pain treatments.

RESULTS

Identifying GPCRs selectively expressed in mouse CGRP$^+$ DRG sensory neurons

Prior transcriptomic and functional analyses revealed physiological and morphological properties of transcriptionally defined somatosensory neurons, including A- and C-fiber low threshold mechanoreceptors (LTMRs), proprioceptors, C-fiber non-peptidergic heat-sensitive high threshold mechanoreceptors (HTMRs), cold thermoreceptors, and six transcriptionally distinct CGRP$^+$ DRG neuron subtypes[5,18] (Figure 1A). At least three CGRP$^+$ DRG neuron subtypes may be considered nociceptors because they respond weakly or not at all to innocuous stimuli and strongly to noxious or painful stimuli.[18,20] Our functional analyses show that the CGRP$^+$ subtypes include two fast-conducting Aδ-HTMR subtypes (*Bmpr1b*$^+$ and *Smr2*$^+$); C-fiber noxious heat-sensing thermoreceptors (*Sstr2*$^+$); C-HTMR/Heat polymodal neurons (*MrgprA3*$^+$); and two subpopulations with unknown physiological functions, CGRP-ε (*Oprk1*$^+$) and CGRP-γ (*Adra2a*$^+$) (Figure 1A).[18,20]

To determine the murine GPCR expression profiles across somatosensory neuron subtypes, a curated list of over 800 non-olfactory GPCR genes[21] was mapped onto our single-cell RNA sequencing (scRNA-seq) dataset.[5] This revealed 69 GPCRs with restricted expression in CGRP$^+$ sensory neuron subtypes, but not in LTMRs or proprioceptors (Table S1), with expression ranging from subtype-specific to broadly expressed across all CGRP$^+$ subtypes. Using the IUPHAR/BPS Guide to Pharmacology as reference, several of these were found to be $G_{i/o}$-coupled, including the serotonin receptor Htr1b (Figure 1B) and α2-adrenergic receptor Adra2a, while others including Gpr19 and Gpr149 are orphan GPCRs with unclear transduction mechanisms (Table S1).

GPCR expression patterns in human DRGs

Toward the long-term goal of translating findings in mice into therapeutic opportunities for humans, we sought to identify GPCRs with conserved expression patterns in mouse and human CGRP$^+$ DRG neurons. *In situ* hybridization (RNAscope) in human DRG tissue showed that *CALCA*, the gene encoding CGRP, was expressed in ∼70% of neurons labeled by *NEFH*, which, unlike the mouse, serves as a pan-neuronal marker for human DRGs[22] (Figures S1A and S1B), consistent with previous findings.[23–27] While CGRP is generally accepted as a nociceptor marker in human DRG studies,[23–27] it is noteworthy that without functional validation it is unclear whether CGRP is a nociceptor marker in human DRGs or whether differences in CGRP expression between mouse and human DRGs are functionally meaningful.

We examined the expression of a subset of candidate GPCR genes in human DRGs, prioritizing known $G_{i/o}$-coupled GPCRs with restrictive expression in mouse CGRP$^+$ neurons and orphan GPCRs with broad CGRP$^+$ subtype expression, reasoning that some orphan receptors may be $G_{i/o}$-coupled. The α2-adrenergic receptor *ADRA2A*, which is highly expressed in one murine CGRP$^+$ subtype, was co-expressed with CGRP in the human DRG (Figures S1C and S1G). The serotonin receptor *HTR1B*, broadly expressed in most murine CGRP$^+$ neuron subtypes including Aδ-HTMRs, C-Heat thermoreceptors, and two CGRP$^+$ subtypes with unknown physiological properties (Figure 1B), exhibited highly overlapping expression with CGRP in the human DRG (Figures 1C and 1D). Additionally, the orphan GPCRs *GPR19*, *GPR35,* and *GPR149* were co-expressed with CGRP in the human DRG (Figures S1D–S1G). Together, we identified GPCRs with similar expression patterns, i.e., co-expression with CGRP, in mouse and human DRGs, along with others with notable differences (Table S1). Candidates exhibiting conserved co-expression with CGRP in both species were prioritized for subsequent screening steps.

DRG GPCRs exhibit distinct G protein coupling

Several orphan GPCRs with unknown G protein transduction mechanisms showed conserved co-expression with CGRP in mouse and human DRGs, including *Gpr19*, *Gpr35*, and *Gpr149*. We used an *in vitro* cAMP assay in a heterologous system to determine whether their human orthologs are $G_{i/o}$-coupled. As a positive control, an α2-adrenergic receptor agonist dexmedetomidine[28] dose-dependently reduced cAMP in *ADRA2A*-expressing, but not vector-expressing, cells (Figure S2A), confirming $G_{i/o}$-coupling specificity.[29] Application of adropin, a putative agonist of GPR19[30,31] (Figure S2B), or kynurenic acid or zaprinast, putative agonists of GPR35[32–34] (Figures S2C and S2D) did not affect cAMP. Pyrrolidine dithiocarbamate (PDTC), a putative agonist of GPR149,[35] reduced cAMP at higher doses in both *GPR149*- and vector-expressing cells (Figure S2E). It remains unclear whether these compounds are *bona fide* agonists or sufficiently potent to activate the receptors in this assay. We therefore exploited ligand-independent constitutive activity, a phenomenon observed when GPCRs are overexpressed,[36–40] to infer signaling mechanisms without prior knowledge of endogenous ligands. Overexpressing the G_s-coupled *ADRB2* significantly elevated cAMP, while overexpression of $G_{i/o}$-coupled *ADRA2A* decreased it, validating this approach (Figure S2F). *GPR19* overexpression inhibited cAMP accumulation, suggesting constitutive $G_{i/o}$-coupling activity (Figure S2G), consistent with previous reports.[30,41] *GPR35* had no effect on cAMP (Figure S2H). *GPR149* overexpression increased cAMP production, suggesting potential G_s-coupling activity (Figure S2I).

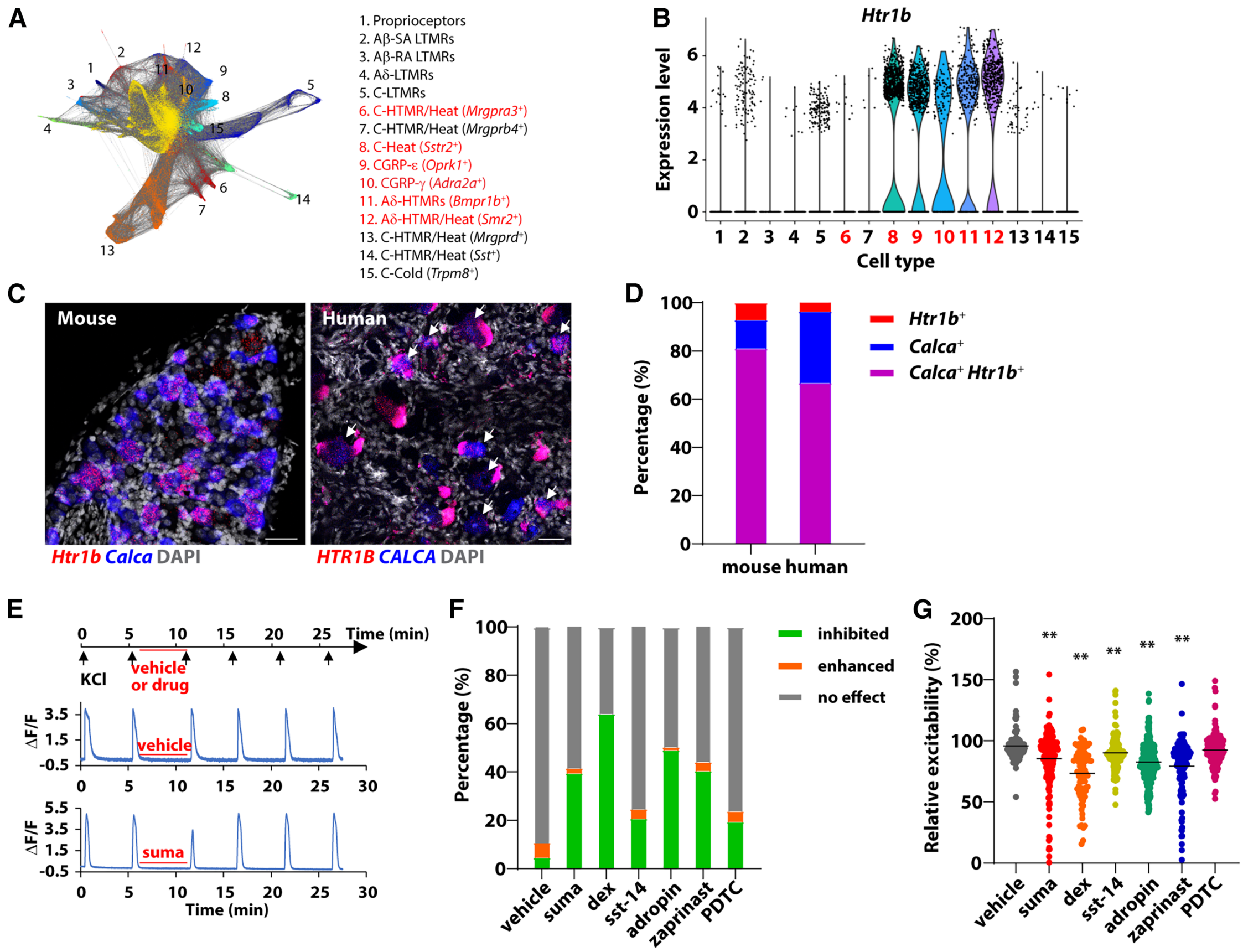

Figure 1. Activating $G_{i/o}$-coupled GPCRs selectively expressed in CGRP⁺ DRG neurons, including the serotonin receptor Htr1b, decreases neuronal excitability

(A) Layout of sensory neuron subtypes in the mouse DRG from our published single-cell RNA sequencing dataset.[5] CGRP⁺ subtypes are highlighted in red.

(B) Expression level of *Htr1b* in the mouse DRG sensory neuron subtypes. The cell types are number coded as in (A).

(C) *In situ* hybridization confirmed co-expression of *Calca* and *Htr1b* in the mouse (left) and human DRGs (right). Human DRG neurons co-expressing *CALCA* and *HTR1B* are indicated by arrows. Scale bars, 50 μm.

(D) Quantification of (C). Percentages of cells that are positive or *Htr1b* only (red), *Calca* only (blue), or *Htr1b* and *Calca* (magenta) among all cells that are positive for at least one marker are plotted.

(E) (Top) Diagram depicting the timeline of calcium imaging. Representative traces of cells treated with vehicle (middle) or sumatriptan (bottom).

(F) A summary plot showing the percentage of cells inhibited or excited by the compounds tested, including vehicle control ($N = 4$), Htr1b/1d agonist sumatriptan (10 μM, $N = 3$); α2-adrenergic receptor agonist dexmedetomidine (dex, 10 μM, $N = 4$); somatostatin receptor agonist Sst-14 (1 μM, $N = 2$); and adropin (100 nM, $N = 4$), zaprinast (30 μM, $N = 3$), and PDTC (10 μM, $N = 3$), putative agonists of Gpr19, Gpr35, and Gpr149 respectively.

(G) The tested compounds showed various degrees of inhibition of neuronal excitability. Each dot represents a cell. Data in this and all other figures are represented as mean ± SEM. All statistical comparisons were performed using a two-tailed Student's *t* test. $^*p < 0.05$, $^{**}p < 0.01$.

Activation of $G_{i/o}$-coupled GPCRs reduces CGRP⁺ DRG neuronal excitability *in vitro*

To determine whether activating $G_{i/o}$-coupled GPCRs inhibits DRG neuron excitability, we utilized an *in vitro* calcium imaging assay in acutely dissociated DRG neurons.[42] GCaMP6f was selectively expressed in CGRP⁺ DRG neurons by crossing *Calca^CreER^* mice[43] with a Cre-dependent reporter (Ai148) and inducing recombination with tamoxifen, and *in vitro* calcium imaging was performed on cultured DRG neurons. KCl-depolarization-induced calcium signaling was used as a surrogate measure for evoked neuronal excitability (Figure 1E, top).[42] While vehicle treatment did not affect KCl-elicited calcium influx (Figure 1E, middle), sumatriptan (10 μM), a selective agonist of the $G_{i/o}$-coupled Htr1b/1d receptors, significantly attenuated the KCl-evoked calcium response (Figure 1E, bottom). Approximately 40% of CGRP⁺ DRG neurons exhibited an attenuated calcium response when pretreated with sumatriptan (Figure 1F). The relative excitability of neurons treated with sumatriptan (magnitude of response normalized to baseline) was lower compared to vehicle-treated control neurons (Figure 1G). Similarly, agonists of other $G_{i/o}$-coupled receptors, including dexmedetomidine (α2 adrenergic receptor agonist) and Sst-14 (somatostatin receptor agonist), attenuated calcium responses in subsets of CGRP⁺ DRG neurons (Figures 1F and 1G). Adropin and zaprinast, putative agonists for Gpr19 and Gpr35 respectively, also decreased KCl-evoked calcium signals, while the putative Gpr149 agonist, PDTC, was without effect (Figures 1F and 1G). Thus, several agonists of $G_{i/o}$-coupled GPCRs or putative

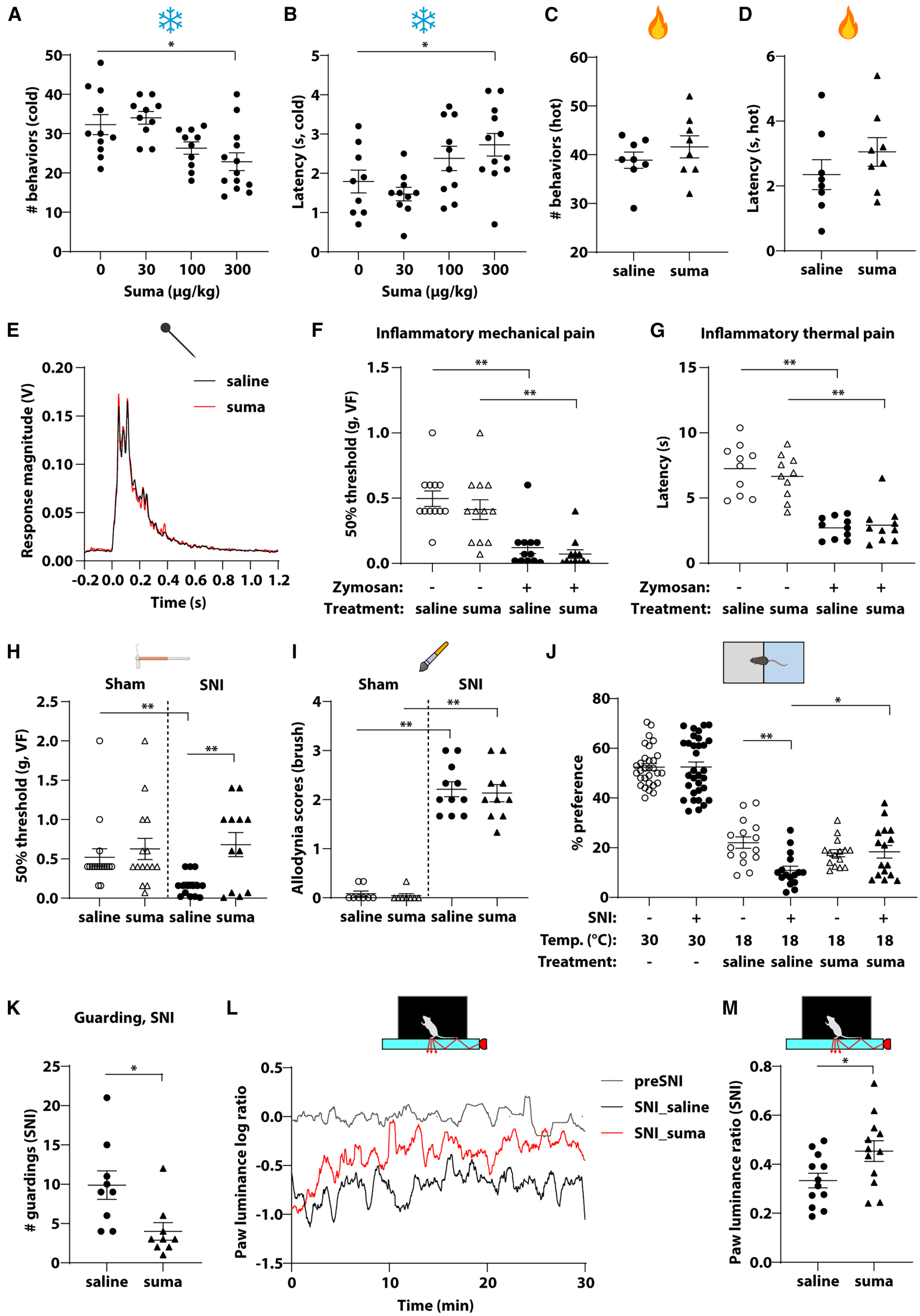

(legend on next page)

$G_{i/o}$-coupled orphan receptors attenuate excitability of subsets of CGRP+ DRG neurons.

CGRP+ DRG neurons are a heterogeneous population including unmyelinated small-diameter C-fiber neurons and myelinated medium- to large-diameter A-fiber neurons (Figure 1A). The calcium imaging findings showed that sumatriptan attenuated excitability of neurons with cell body diameters ranging from small to large (Figure S3A, left), consistent with the broad expression of *Htr1b* in C- and A-fiber CGRP+ neurons (Figure 1B). Notably, sumatriptan-attenuated neurons had larger cell body diameters than sumatriptan-insensitive neurons (Figure S3A, left). While ~30% of small-diameter (<25 μm) neurons were inhibited by sumatriptan, over 40% of medium- (25–35 μm) and 65% of large-diameter (>35 μm) neurons were inhibited (Figure S3A, right). These findings suggest that although sumatriptan can act on both C- and A-fiber CGRP+ neurons, medium- to large-diameter A-fiber CGRP+ neurons may be more responsive. In contrast, dexmedetomidine and Sst-14 mostly inhibited small diameter neurons (Figures S3B and S3C), consistent with *Adra2a* and *Sstr2* expression in small-diameter C-fiber CGRP+ neurons (Figures 1A and S1C, left). We did not observe a clear trend of cell diameters for the neurons responding to adropin or zaprinast, the putative agonists of Gpr19 and Gpr35, respectively (Figures S3D–S3F).

Attenuation of behavioral responses to noxious cold by sumatriptan

We next focused on Htr1b, a well-characterized $G_{i/o}$-coupled receptor identified in our screen, and its agonists, the anti-migraine drug triptans, as a proof-of-principle to test the hypothesis that activating nociceptor-specific $G_{i/o}$-coupled GPCRs leads to silencing of nociceptors and attenuation of nociception. A focus on Htr1b may also help reveal the site of action of triptans and whether they can be used for indications other than migraine.

The triptans are prescribed for migraine and cluster headaches with limited clinical testing in other pain conditions.[14] However, *Htr1b*, as we have shown, is broadly expressed in CGRP+ DRG sensory neurons across all axial levels rather than limited to the trigeminal system, and our *in vitro* findings suggest that sumatriptan reduces nociceptor excitability. We therefore systematically tested the analgesic potential of sumatriptan across a battery of pain behavior assays in mice. In the cold plate assay, sumatriptan reduced nocifensive behavioral responses at a dose of 300 μg/kg, delivered intraperitoneally (i.p.), which is comparable to the dose clinically used in human patients[44] (Figures 2A and 2B). Sumatriptan pretreatment led to a reduction in the number of behavioral bouts in response to noxious cold and an increase in the latency to the first reactive behavior (Figures 2A and 2B). In contrast, sumatriptan was ineffective in reducing noxious heat responsiveness in a hot plate assay (Figures 2C and 2D), acute mechanical pain in a pinprick assay (Figures 2E, S4A, and S4B), or inflammatory mechanical and thermal pain induced by intraplantar injection of zymosan (Figures 2F and 2G).

Attenuation of neuropathic pain by sumatriptan

We next asked whether sumatriptan is effective in neuropathic pain. Although the effects of sumatriptan in non-cranial neuropathic pain have not been systematically investigated clinically, preclinical studies have shown mixed results.[45–48] We used the spared nerve injury (SNI) model, which induces a robust and persistent form of neuropathic pain.[49,50] Static/punctate allodynia and dynamic mechanical allodynia were tested using von Frey (VF) filaments and dynamic brush, respectively. Sumatriptan reduced punctate mechanical allodynia in the VF assay (Figure 2H), but not dynamic brush allodynia (Figures 2I and S4C).

To assess cold hypersensitivity, we used a temperature preference assay in which animals could freely move between floor surfaces set at 30°C/30°C or 30°C/18°C. No side bias was observed before or after surgery when both sides of the floor were set at 30°C. When given a choice between floor temperature of 30°C or 18°C, mice exhibited a preference for 30°C, and SNI surgery conferred a more pronounced aversion to 18°C (Figure 2J), suggesting cold hypersensitivity. Notably, sumatriptan did not alter temperature sensitivity in non-SNI mice but restored the cold preference of SNI mice to pre-SNI levels (Figure 2J).

Two weeks after SNI, mice exhibited a spontaneous guarding behavior on the ipsilateral hind paw. This behavior was observed during rest in the absence of external triggers, suggesting spontaneous pain. The guarding behavior was attenuated by gabapentin, a medication commonly prescribed to treat neuropathic pain[51] (Figure S4D), suggesting it reflects aberrant sensory

Figure 2. Sumatriptan attenuates responses to noxious cold and SNI-induced neuropathic pain

(A and B) Sumatriptan (30–300 μg/kg, i.p.) dose-dependently reduced nocifensive behaviors (A) and increased the latency to the first nocifensive behavior (B) in the cold plate (0°C) assay. The dose of 300 μg/kg was used for all other behavior tests unless otherwise specified.

(C and D) Sumatriptan did not affect the number of behaviors (C) or latency (D) in the hot plate (51°C) assay.

(E) Sumatriptan had no effect in reducing noxious mechanical pain in the pinprick assay. Averaged response traces of saline- (black) and sumatriptan-treated (red) animals were shown. Time 0 indicates the onset of the pinprick stimulus.

(F) Inflammatory mechanical pain induced by intraplantar injection of zymosan was assessed by the von Frey (VF) test. Decrease of mechanical threshold after zymosan treatment was not alleviated by sumatriptan.

(G) Sumatriptan had no effect on zymosan-induced thermal hyperalgesia in the Hargreaves test.

(H) Sumatriptan alleviated punctate mechanical allodynia after SNI surgery in the VF test.

(I) Sumatriptan did not affect the allodynia score in the dynamic brush test (C).

(J) Sumatriptan reversed SNI-induced cold hypersensitivity in the temperature preference assay.

(K) Sumatriptan also attenuated guarding behaviors in SNI animals.

(L) Representative traces of the paw luminance log ratio [calculated as logarithmic ($\frac{Luminance\_ipsilateral\_hind\_paw}{Luminance\_contralateral\_hind\_paw}$)] of preSNI (gray) and saline- (black) or sumatriptan (red)-treated SNI animals in the paw luminance assay.

(M) Sumatriptan partially reversed the reduction of paw luminance ratio after SNI surgery.

rather than motor function. Sumatriptan similarly reduced guarding behavior in SNI mice (Figure 2K).

To further assess sumatriptan's effect on spontaneous pain, we employed a paw luminance assay that measures paw surface contact via frustrated total internal reflection technology (FTIR) and simultaneously records the whole-body pose.[52] SNI mice exhibited a reduced paw luminance ratio (ipsilateral to contralateral hind paw), reflecting reduced weight bearing on the injured limb (Figures 2L and S4E). Sumatriptan attenuated SNI-induced reduction of paw luminance ratio in both locomoting and non-locomoting states (Figures 2L, 2M, and S4E–S4G). Concurrent whole-body pose recording confirmed that sumatriptan did not affect travel distance, locomotion time, or speed (Figures S4H–S4J), ruling out an effect on motor behavior. Moreover, the ipsilateral hind paw angle and the inter-hind-paw distance were not affected by sumatriptan (Figures S4K and S4L), suggesting that sumatriptan did not alter gait. Therefore, it is unlikely that the increase of the luminance ratio observed in sumatriptan-treated mice was due to altered ambulatory behavior or gait.

Collectively, these findings suggest that sumatriptan confers analgesia in somatic pain models. Sumatriptan reduced responses to noxious cold under normal conditions but was ineffective in noxious heat, mechanical, and inflammatory pain assays (Figures 2A–2G). Strikingly, in the SNI model, sumatriptan attenuated static mechanical allodynia, cold hypersensitivity, and two measures of spontaneous pain (Figures 2H–2M).

Sumatriptan acting through Htr1b reduces both CGRP$^+$ neuron excitability and synaptic transmission in the spinal cord

We next sought to determine the potential sites of action of sumatriptan. Sumatriptan is a potent agonist for both Htr1b and Htr1d but exhibits a much lower affinity for the other serotonin receptors.[53–55] Our sequencing data suggested that Htr1d is expressed at low levels in proprioceptors and C- and Aδ-LTMRs (Figure S5A), while Htr1b is exclusively and highly expressed in CGRP$^+$ DRG neurons (Figure 1B). We confirmed by RNAscope that *Htr1d* and *Htr1b* are expressed in a non-overlapping manner in both mouse and human DRG (Figures S5B and S5C). Since GCaMP6f was exclusively expressed in CGRP$^+$ neurons in our *in vitro* calcium imaging assay, we speculated that the inhibitory effect of sumatriptan is mediated through Htr1b. To test this, we generated conditional knockout (cKO) mice using *Calca*^{CreER} and a Cre-dependent *Htr1b* conditional allele (*Htr1b*^{fl})[56] to selectively delete *Htr1b* in CGRP$^+$ neurons that express GCaMP6f (*Calca*^{CreER}*; Ai95; Htr1b*$^{fl/fl}$, hereafter denoted *Htr1b* cKO^{Calca}). Efficient deletion of *Htr1b* in CGRP$^+$ DRG neurons was confirmed by RNAscope (Figures S5D and S5E). In *Htr1b* cKO^{Calca} mice, sumatriptan failed to attenuate excitability of CGRP$^+$ neurons (Figures 3A and 3B), indicating that Htr1b mediates the inhibitory effect of sumatriptan on CGRP$^+$ DRG neuron excitability.

The *in vitro* calcium imaging experiments indicate that sumatriptan attenuates neuronal excitability when applied directly to CGRP$^+$ DRG neurons. *In vivo*, sumatriptan could act on cell bodies, peripheral axons, and/or central axon terminals of these sensory neurons. As a relatively hydrophilic compound, sumatriptan was initially considered unable to cross the blood-brain barrier (BBB). However, recent findings have suggested that sumatriptan can cross the BBB and activate Htr1b/1d receptors in the central nervous system.[57–60] To explore the possibility of a central site of action of sumatriptan in blocking nociceptor function, we performed *ex vivo* slice electrophysiology in the spinal cord. We used *Na*$_v$*1.8*^{Cre}, which labels all CGRP$^+$ neurons and additional C-fiber neuron subtypes,[61,62] Cre-dependent channelrhodopsin (Ai32), and the *Htr1b*^{fl} allele, to express the photoactivatable opsin ChR2 (*Na*$_v$*1.8*^{ChR2}) in *Htr1b*$^{+/+}$ and *Htr1b* cKO$^{Nav1.8}$ (*Na*$_v$*1.8*^{Cre}*; Ai32; Htr1b*$^{fl/fl}$) mice. We prepared acute lumbar spinal cord slices from *Htr1b*$^{+/+}$ and *Htr1b* cKO$^{Nav1.8}$ mice and made whole-cell patch-clamp recordings from neurons within lamina I-II$_o$ of the dorsal horn, where peptidergic C-fiber DRG neurons and some Aδ-HTMR fibers terminate[18,20] (Figure 3C). Excitatory postsynaptic currents (EPSCs) were evoked by optogenetically stimulating *Na*$_v$*1.8*^{ChR2} primary afferent axonal terminals. We observed a significant reduction of optically evoked EPSC amplitudes (oEPSCs) following sumatriptan application in *Htr1b*$^{+/+}$ wild-type animals (Figures 3D and 3E). In *Htr1b* cKO$^{Nav1.8}$ mice (*Na*$_v$*1.8*^{Cre}*; Ai32; Htr1b*$^{fl/fl}$), in which *Htr1b* was efficiently depleted (Figures S5F and S5G), the attenuation of the oEPSC amplitude by sumatriptan observed in the *Htr1b*$^{+/+}$ control littermates was abolished (Figures 3F and 3G), indicating that sumatriptan can act presynaptically through Htr1b in sensory neurons to reduce synaptic transmission. This finding is consistent with previous findings that sumatriptan can inhibit synaptic neurotransmission in trigeminal sensory neurons.[63–65]

Generating and characterizing mice lacking Htr1b in adult somatosensory neurons

The *in vitro* and *ex vivo* findings suggest that Htr1b expressed in sensory neurons is responsible for triptan-mediated inhibition of CGRP$^+$ DRG neuron excitability and suppression of presynaptic neurotransmission in the spinal cord. To determine whether the analgesic effects of sumatriptan are mediated by Htr1b and/or Htr1d or other target(s), and whether somatosensory neurons are its primary site of action, we generated mice that lack Htr1b receptors in all somatosensory neurons using a pan-somatosensory neuron CreER driver line (*Avil*^{CreER}*; Htr1b*$^{fl/fl}$, herein denoted *Htr1b* cKO^{Avil}).[66] CreER-mediated recombination was induced by exposing young adult mice (P28-32) to tamoxifen (1 mg/day for 5 days) to excise *Htr1b* in somatosensory neurons. Knockout efficiency in DRG neurons was ∼74%, as determined by RNAscope (Figures S6A and S6B).

The pan-sensory neuron *Htr1b* cKO^{Avil} mice appeared grossly normal compared to control littermates, with comparable body size, weight, and viability. They also exhibited normal locomotor activity in the open field (OF) test (Figures S6C and S6D). Mechanical and temperature sensitivity of *Htr1b* cKO^{Avil} mice were also comparable to their control littermates (Figures S6E and S6F). In a sandpaper avoidance behavioral test, while control animals consistently avoided a rough texture, *Htr1b* cKO^{Avil} mice lost this preference (Figure S6G).

Analgesic actions of sumatriptan are mediated through Htr1b expressed in somatosensory neurons

We used the *Htr1b* cKO^{Avil} mice to test whether sumatriptan acts on Htr1b in somatosensory neurons to promote its analgesic

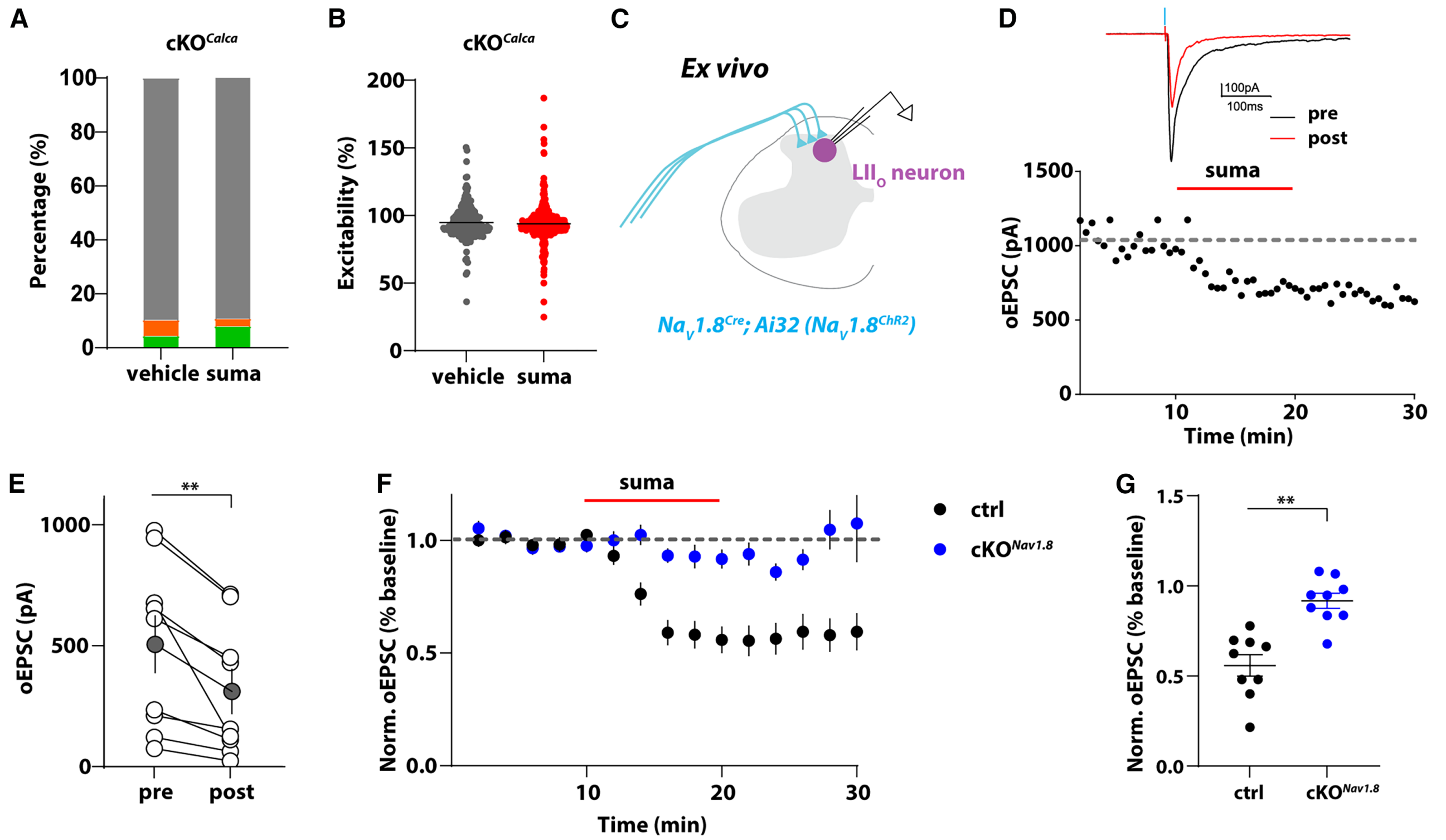

Figure 3. Htr1b in sensory neurons is responsible for inhibition of CGRP⁺ DRG neurons *in vitro* and suppression of glutamate release from sensory neuron synapses in the spinal cord dorsal horn

(A) $CGRP^+$ cells no longer respond to sumatriptan in *Htr1b* cKO^{Calca} animals ($N = 3$).

(B) Vehicle- and sumatriptan-treated cells from *Htr1b* cKO^{Calca} animals show comparable excitability.

(C) Schematic diagram of the slice electrophysiology experiment. ChR2 was expressed in $CGRP^+$ and other C-fiber sensory neuron subtypes using $Na_v1.8^{Cre}$ and the Cre-dependent reporter Ai32.

(D) Representative response of a lamina II dorsal horn neuron to sumatriptan. Sumatriptan application decreased the amplitude of optically evoked EPSC (oEPSC).

(E) Quantification of the oEPSCs before and after sumatriptan treatment ($N = 3$, $n = 9$). Each dot represents one neuron; gray dots show average oEPSC values.

(F) Sumatriptan-mediated inhibition of oEPSC inhibition in control mice (black, $N = 3$, $n = 9$) was diminished in *Htr1b* $cKO^{Nav1.8}$ littermates (blue, $N = 4$, $n = 9$).

(G) Quantification of (F).

effects. Indeed, in the noxious cold plate assay, sumatriptan reduced nocifensive behaviors in control animals (Figures 4A and 4B), as predicted from our prior findings (Figures 2A and 2B); however, it failed to do so in *Htr1b* cKO^{Avil} mice (Figures 4A and 4B). Both the magnitude and latency of responses to noxious cold were unaltered by sumatriptan in *Htr1b* cKO^{Avil} mice. Similarly, in the SNI model of neuropathic pain, sumatriptan reduced the guarding behaviors in control but not *Htr1b* cKO^{Avil} animals (Figure 4C). These findings indicate that the analgesic effects of sumatriptan are mediated by Htr1b receptors expressed in the sensory neurons.

As *Htr1b* is broadly expressed in several transcriptionally and functionally distinct subtypes of $CGRP^+$ sensory neurons (Figure 1B), we next sought to determine which of the subtypes are important for sumatriptan-mediated analgesia. Although the C-fiber $CGRP^+$ neuron subtypes are traditionally considered "nociceptors," we have found that the Aδ-HTMR subtypes ($Bmpr1b^+$ or $Smr2^+$) are fast conducting neurons that respond most robustly to noxious mechanical stimuli and thus may be classified as myelinated nociceptors.[18,20] In an *in vivo* calcium imaging assay in which GCaMP was specifically expressed in $Bmpr1b^+$ or $Smr2^+$ DRG neurons ($Bmpr1b^{Cre}$; $Calca^{FlpE}$[67]; *Ai195* or $Smr2^{Cre}$; *Ai148*, respectively), a subset of $Bmpr1b^+$ or $Smr2^+$ neurons responded to the noxious cold stimuli (0°C) delivered to the glabrous skin of the hind paw (Figures S6H and S6I), implicating these neurons as mediators of noxious cold pain. We then generated $Bmpr1b^{Cre}$; $Htr1b^{fl/fl}$ (cKO^{Bmpr1b}), $Smr2^{Cre}$; $Htr1b^{fl/fl}$ (cKO^{Smr2}), and $Bmpr1b^{Cre}$; $Smr2^{Cre}$; $Htr1b^{fl/fl}$ ($cKO^{Bmpr1b; Smr2}$) mice, which lack *Htr1b* in either $Bmpr1b^+$, $Smr2^+$, or both $Bmpr1b^+$ and $Smr2^+$ A-fiber HTMR DRG neurons (Figures S6J and S6K). The cKO^{Bmpr1b}, cKO^{Smr2}, and $cKO^{Bmpr1b; Smr2}$ animals behaved grossly normal and showed comparable locomotor activity to control littermates (Figures S6L and S6M).

We performed the cold plate assay on the single- or double Aδ-HTMR subtype *Htr1b* cKO mice and their control littermates treated with either saline or sumatriptan. Again, sumatriptan reduced nocifensive behaviors in control mice but not in *Htr1b* Aδ-HTMR cKO mice (Figure 4D). Sumatriptan increased the behavioral latency in *Htr1b* cKO^{Bmpr1b} mice and showed a non-significant trend toward increased latency in *Htr1b* cKO^{Smr2}

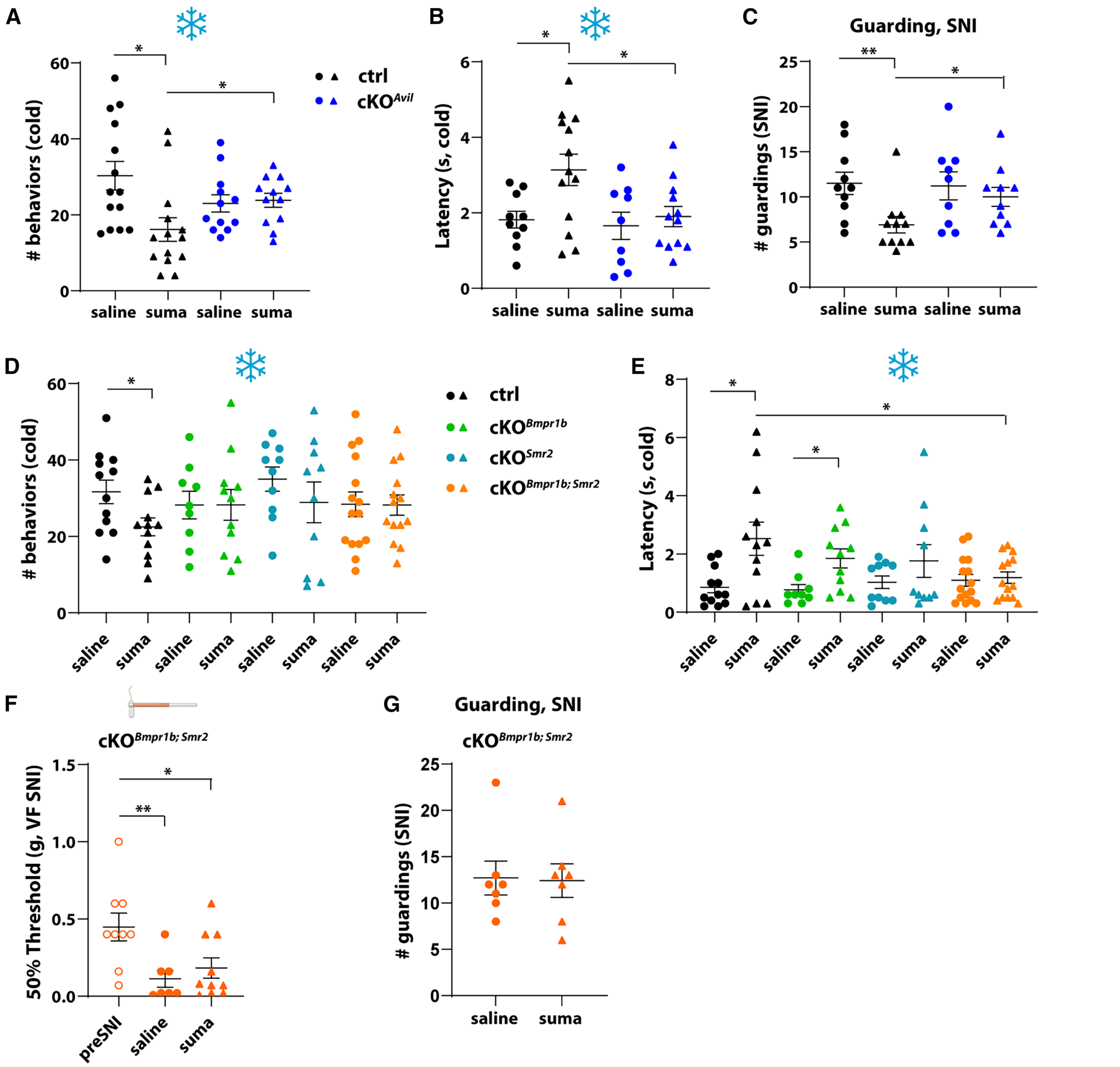

Figure 4. The analgesic effect of sumatriptan is mediated by Htr1b in sensory neurons, particularly the *Bmpr1b*⁺ and *Smr2*⁺ Aδ-HTMR subpopulations

(A and B) In the cold plate assay, the sumatriptan-mediated reduction of nocifensive behaviors (A) and the increase of latency (B) in control mice were abolished in the *Htr1b* cKO^{Avil} mice.

(C) Sumatriptan reduced guarding behaviors in control mice but not in *Htr1b* cKO ^{Avil} mice after SNI surgery.

(D and E) In the cold plate assay, the analgesic effect of sumatriptan was partially diminished in the *Htr1b* cKO ^{Bmpr1b} or cKO ^{Smr2} mice and completely abolished in the cKO $^{Bmpr1b;\ Smr2}$ animals.

(F) SNI-induced mechanical allodynia, reflected by reduced 50% threshold in the VF test, was not alleviated by sumatriptan in the *Htr1b* cKO $^{Bmpr1b;\ Smr2}$ animals.

(G) Sumatriptan failed to reduce guarding behaviors in the *Htr1b* cKO $^{Bmpr1b;\ Smr2}$ animals after SNI surgery.

mice (Figure 4E). The double Aδ-HTMR subtype cKO mice (*Htr1b* cKO$^{Bmpr1b;\ Smr2}$) were insensitive to sumatriptan, exhibiting unaffected nocifensive behaviors and latency (Figures 4D and 4E). Given the similar functional properties of both subtypes, we used *Htr1b* cKO$^{Bmpr1b;\ Smr2}$ mice, which lack Htr1b in both Aδ-HTMR subtypes, for subsequent behavioral testing.

We next performed SNI surgery on cKO$^{Bmpr1b;\ Smr2}$ mice to induce neuropathic pain. Remarkably, sumatriptan failed to attenuate static mechanical allodynia and the persistent guarding behavior in *Htr1b* cKO$^{Bmpr1b;\ Smr2}$ mice (Figures 4F and 4G), in contrast to its analgesic effects in wild-type animals (Figures 2H, 2K, and 4C). These findings indicate that Htr1b in somatosensory neurons, particularly the large diameter *Bmpr1b*⁺ and *Smr2*⁺ Aδ-HTMRs, is the principal target of sumatriptan for attenuating pain. This conclusion is consistent with our *in vitro* neuronal excitability measurements (Figure 1), showing

CellPress
OPEN ACCESS

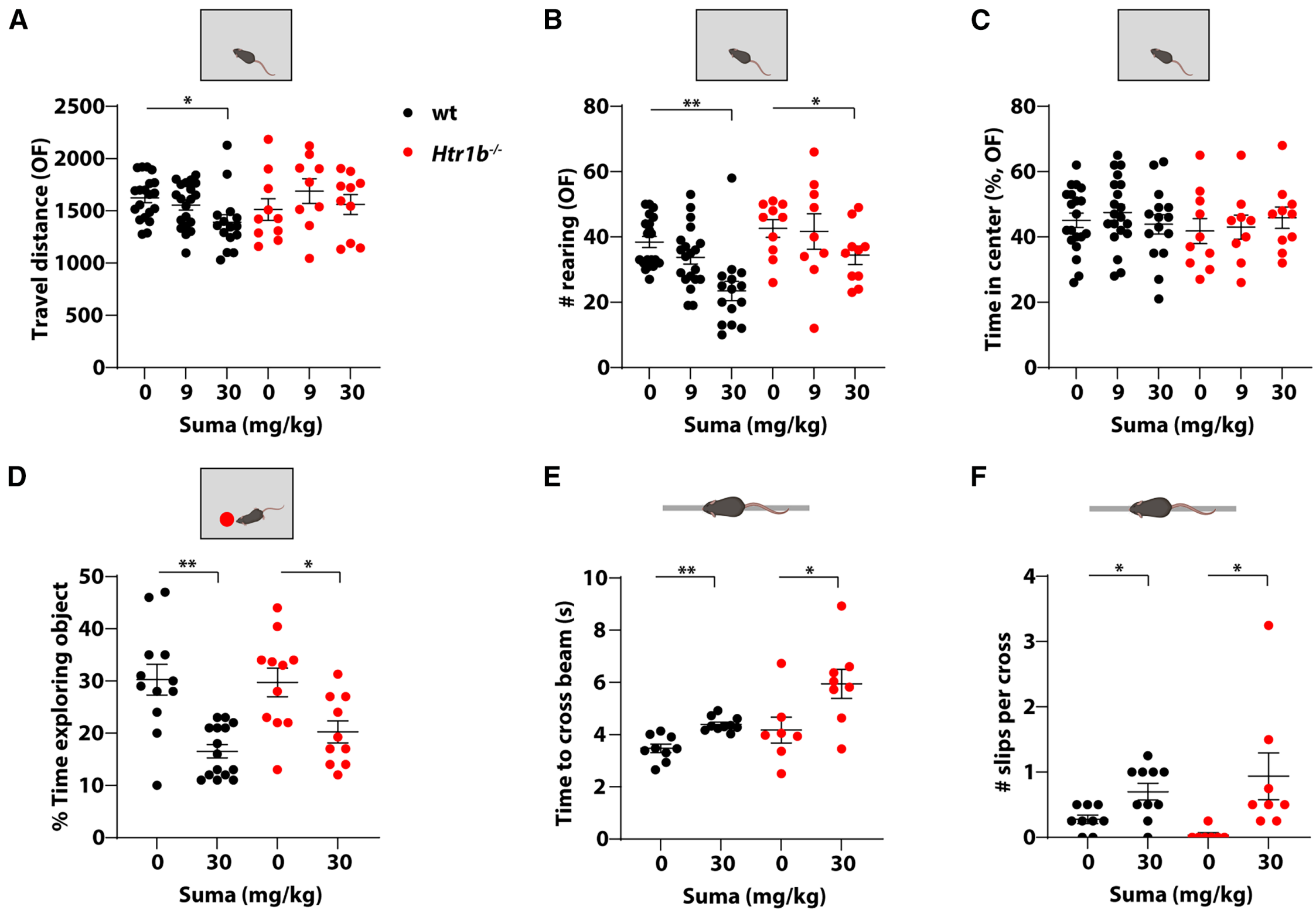

Figure 5. Adverse effects associated with a high dose of sumatriptan are at least partially due to Htr1b-independent off-target effects

(A–C) A high dose of sumatriptan (30 mg/kg) reduced ambulatory activities in the open field assay. The travel distance was slightly reduced in wild-type (wt) but not *Htr1b*$^{-/-}$ mice (A), and rearing behaviors were significantly reduced in both wt and *Htr1b*$^{-/-}$ mice (B), while the time spent in the center of the chamber was not affected (C).

(D) When given a high dose of sumatriptan, both wt and *Htr1b*$^{-/-}$ mice spent much less time exploring a novel object.

(E and F) Both wt and *Htr1b*$^{-/-}$ mice exhibited impaired performance in a balance beam test when given a high dose of sumatriptan, demonstrating longer crossing time (E) and more slips during the crossing (F).

that large-diameter CGRP$^+$ neurons are more sensitive to sumatriptan's effects on attenuating excitability compared to small-diameter CGRP$^+$ DRG neurons (Figure S3A).

Htr1b-dependent and -independent adverse side effects of high-dose sumatriptan

Although triptans including sumatriptan have been the first-line treatment for migraine, there are common adverse side effects associated with triptans including dyspnea, dizziness, paresthesia, and chest pain or tightness, which limit their clinical utility.[68–70] We therefore assessed sumatriptan-associated adverse effects in mice to determine whether they are mediated by Htr1b.

Preclinical studies have shown that rats receiving subcutaneous sumatriptan at a dose of 12.5 mg/kg did not exhibit overt adverse effects, while 25 mg/kg caused moderate behavioral depression.[71] When sumatriptan was given to mice at a dose of 30 mg/kg, which is 100 times greater than the dose that conferred analgesia (300 μg/kg), animals exhibited normal heart rate (Figure S7A), consistent with prior preclinical findings that sumatriptan is minimally vasoactive outside the trigeminal system.[71,72] This high dose of sumatriptan reduced ambulatory activity in mice, as inferred from slightly reduced travel distance and substantially reduced rearing behaviors in the OF test (Figures 5A and 5B). The time animals spent in the center of the chamber, grooming, or motionless was not different between saline- and high-dose-sumatriptan-treated groups (Figures 5C, S7B, and S7C), suggesting that the high dose of sumatriptan did not cause anxiety or sedation. In a novel object exploration test, mice receiving a high dose of sumatriptan spent considerably less time exploring a novel object (Figure 5D). In the balance beam test, high-dose sumatriptan-treated mice required more time to cross the beam and slipped more often while crossing (Figures 5E and 5F). These findings suggest that a high dose of sumatriptan caused moderately compromised well-being, manifesting in reduced ambulatory activity and rearing, reduced exploration of a novel object, and moderately impaired balance.

To ask whether Htr1b is responsible for these adverse effects, we generated constitutive *Htr1b* knockout mice (*Htr1b*$^{-/-}$) by excising the *Htr1b* gene from the *Htr1b*^{fl} allele using a germline Cre driver, EIIa-Cre.[73] *Htr1b* expression is completely depleted in *Htr1b*$^{-/-}$ mice (Figure S7D). These animals behaved grossly normally in the OF test compared to control mice, exhibiting comparable travel distance, rearing behaviors, time in the center of chamber, or motionless time (Figures 5A–5C and S7C),

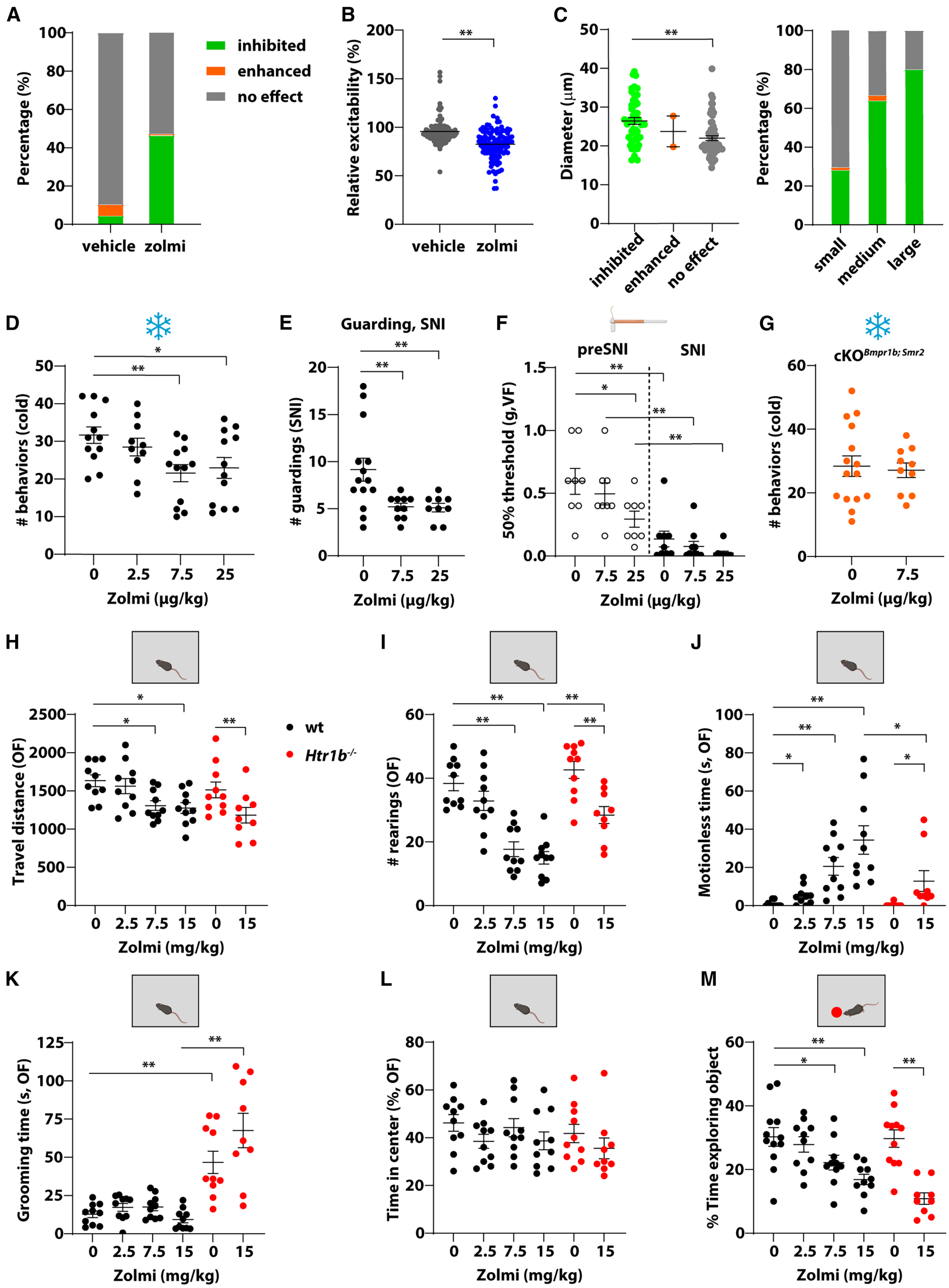

Figure 6. Zolmitriptan attenuates neuronal excitability and exhibits comparable analgesic effects to sumatriptan in an Htr1b-dependent manner, although it exhibits more severe adverse effects at high doses

(A) Over 40% of CGRP$^+$ neurons were inhibited by zolmitriptan (10 μM) in the *in vitro* calcium imaging assay ($N = 3$).

(B) Zolmitriptan attenuated excitability of CGRP$^+$ neurons.

(C) Large-diameter CGRP$^+$ neurons were more responsive to zolmitriptan.

(legend continued on next page)

although *Htr1b*$^{-/-}$ mice did spend more time grooming (Figure S7B).

When given a high dose of sumatriptan, *Htr1b*$^{-/-}$ mice traveled comparable distances to saline-treated *Htr1b*$^{-/-}$ mice (Figure 5A) and spent a similar amount of time in the center (Figure 5C). However, high-dose sumatriptan-treated *Htr1b*$^{-/-}$ mice showed reduced rearing behaviors compared to the saline-treated *Htr1b*$^{-/-}$ controls (Figure 5B). The high dose of sumatriptan also significantly reduced exploration of a novel object in *Htr1b*$^{-/-}$ mice (Figure 5D). Moreover, sumatriptan-treated *Htr1b*$^{-/-}$ mice performed less proficiently in the balance beam test, exhibiting a longer crossing time with more slips during crossing (Figures 5E and 5F). We conclude that many of the adverse effects associated with a high dose of sumatriptan are due to target(s) other than Htr1b. Considering that triptans are Htr1b/1d-selective agonists, we speculate that Htr1d may be the "off-target," although we cannot rule out the possibility that other serotonin receptors, despite having a much lower affinity, or some other unknown target, underlie these effects.

Another triptan, zolmitriptan, acts via Htr1b in somatosensory neurons to reduce pain and exhibits Htr1b-independent adverse effects

To further validate our findings with a second triptan, we tested zolmitriptan, which unlike sumatriptan is lipophilic and has a longer half-life and higher oral bioavailability.[74] We assessed its analgesic efficacy in a subset of behavioral assays and whether its site of action, like sumatriptan, is Htr1b in sensory neurons.

Zolmitriptan (10 μM) inhibited excitability of CGRP$^+$ neurons *in vitro* to a similar extent as sumatriptan (Figures 6A and 6B), with a comparable cell size distribution of responding neurons (Figure 6C). In behavioral assays, zolmitriptan attenuated noxious cold pain at a much lower dose (7.5–25 μg/kg) than sumatriptan (300 μg/kg), demonstrating higher potency but comparable efficacy (Figure 6D). In the SNI model, zolmitriptan alleviated spontaneous pain (Figure 6E) but, unlike sumatriptan, did not affect static mechanical allodynia (Figure 6F). Notably, zolmitriptan slightly reduced mechanical thresholds in preSNI animals (Figure 6F), an effect not observed with the more peripherally restricted sumatriptan (Figures 2F and 2H), suggesting a central action that may mask any analgesic effect mediated by Htr1b in sensory neurons and may also account for the more severe adverse effects of zolmitriptan compared to sumatriptan (Figures 6H–6M). The analgesic effect of zolmitriptan in the cold plate assay was abolished by deleting *Htr1b* in both *Bmpr1b*$^+$ and *Smr2*$^+$ neurons (Figure 6G), suggesting that it is mediated by Htr1b in Aδ-HTMRs.

At doses of 7.5 mg/kg or higher, zolmitriptan reduced travel distance and rearing behaviors in the OF test (Figures 6H and 6I). High-dose-zolmitriptan-treated animals appeared subdued and less active, displaying increased motionless time even at 2.5 mg/kg and plateauing at 15 mg/kg (Figure 6J), while grooming or time in the center was unaffected (Figures 6K and 6L). Zolmitriptan also dose-dependently reduced novel object exploration (Figure 6M). Overall, zolmitriptan produced similar but more severe adverse effects than sumatriptan, even at lower doses. In *Htr1b*$^{-/-}$ mice, a high dose of zolmitriptan similarly reduced travel distance, rearing behaviors, and novel object exploration and increased motionless time, though the latter was less severe than in wild-type mice (Figures 6H–6M). As with sumatriptan, zolmitriptan-associated adverse effects are therefore largely Htr1b-independent. The finding that some zolmitriptan-triggered adverse behaviors in *Htr1b*$^{-/-}$ mice were less severe than those in wild-type mice suggests that at least some adverse effects are associated with Htr1b, possibly due to a central action of zolmitriptan.

Gpr19, an orphan GPCR, is a promising candidate target for treating pain

Several orphan GPCRs emerge from our screening as interesting candidates, including Gpr19, which exhibits co-expression with CGRP in both mouse and human DRGs (Figures S1D and S1G) and constitutive $G_{i/o}$-coupling activity *in vitro* (Figure S2G). Moreover, the putative Gpr19 agonist, adropin, inhibits excitability of CGRP$^+$ DRG neurons *in vitro* (Figures 1F and 1G). To ask whether Gpr19 is the target of adropin in inhibiting CGRP$^+$ neurons, we generated *Gpr19* cKO^{Calca} mice (*Calca*^{CreER}*; Ai148; Gpr19*$^{fl/fl}$) and confirmed efficient depletion of *Gpr19* using *in situ* hybridization (Figures 7A and 7B). In the *in vitro* calcium imaging assay, the inhibitory effects of adropin on CGRP$^+$ neuron excitability were diminished but not abolished in neurons obtained from *Gpr19* cKO^{Calca} mice (Figures 7C and 7D), suggesting that adropin acts at least partially through Gpr19 to inhibit neuronal excitability. Although *Gpr19* is mainly expressed in C-fiber neurons in the mouse DRG (Figure S1D, left), adropin inhibited small-, medium-, and large-diameter neurons comparably (Figure S3D), further implicating both Gpr19 and non-Gpr19 sites of adropin action.

In vivo, adropin moderately attenuated nocifensive responses to noxious heat (Figure 7E) but not noxious cold (Figure 7F), consistent with *Gpr19* expression in the C-Heat sensory neuron subtype marked by *Sstr2* (Figure S1D, left). In the SNI-induced neuropathic pain model, adropin had no effect on mechanical allodynia (Figure 7G), cold hypersensitivity (Figure 7H), or the spontaneous pain-like guarding behavior (Figure 7I). To determine the site of action of adropin, we generated *Gpr19* cKO^{Avil} mice, in which *Gpr19* was specifically depleted in peripheral nervous system neurons including DRG neurons (*Avil*^{CreER}*; Gpr19*$^{fl/fl}$; Figures 7A and 7B). Surprisingly, the *Gpr19* cKO^{Avil}

(D) Zolmitriptan dose-dependently attenuated noxious cold pain in the cold plate test. (E) Zolmitriptan reduced guarding behaviors in SNI mice.
(F) Zolmitriptan did not affect SNI-induced mechanical allodynia.
(G) The analgesic effect of zolmitriptan in noxious cold was lost in the *Htr1b* cKO$^{Bmpr1b; Smr2}$ animals.
(H–L) In the open field assay, zolmitriptan dose-dependently reduced ambulatory activities of both wt and *Htr1b*$^{-/-}$ mice. Travel distance (H) and rearing behaviors (I) were reduced after high-dose zolmitriptan treatment. High doses of zolmitriptan also triggered a motionless state in both wt and *Htr1b*$^{-/-}$ mice that resembles sedation (J). Grooming (K) and time in the center (L) was not affected by the high doses of zolmitriptan.
(M) High doses of zolmitriptan significantly reduced novel object exploring in both wt and *Htr1b*$^{-/-}$ mice.

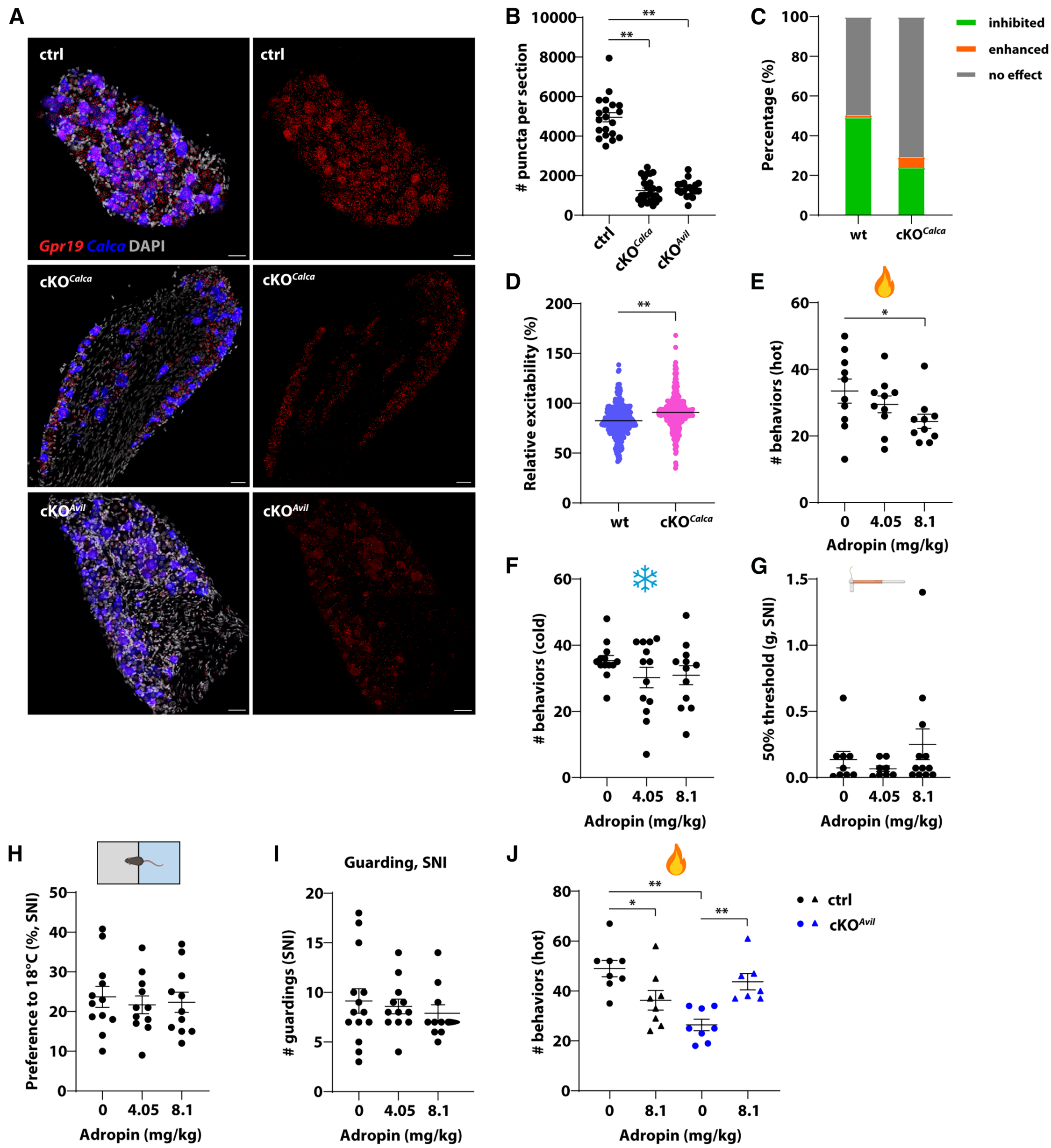

Figure 7. Gpr19 is a potential drug target for treating pain

(A) RNAscope confirmed efficient deletion of *Gpr19* in the DRG using either *Calca*^{CreER} or *Avil*^{CreER}. Scale bars, 50 μm.

(B) Quantification of (A). $N = 3$ for each genotype.

(C) The percentage of CGRP$^+$ neurons inhibited by adropin (100 nM) was reduced in *Gpr19* cKO^{Calca} mice (*Calca*^{CreER}*; Ai148; Gpr19*$^{fl/fl}$) compared to control mice.

(D) The average relative excitability of adropin-treated CGRP$^+$ neurons was higher in *Gpr19* cKO^{Calca} mice than controls.

(E) Adropin dose-dependently reduced noxious-heat-induced nocifensive behaviors.

(F) Adropin did not affect acute noxious cold pain.

(G–I) Adropin had no effect on SNI-induced mechanical allodynia (G), cold hypersensitivity (H), or guarding behavior (I).

(J) Adropin (8.1 mg/kg) attenuated noxious heat pain in control mice but augmented nocifensive behaviors in *Gpr19* cKO^{Avil} mice (*Avil*^{CreER}*; Gpr19*$^{fl/fl}$).

mice exhibited hyposensitivity to heat compared to control littermates (Figure 7J). This may suggest an unknown role of Gpr19 in noxious heat detection, although the underlying mechanism is unclear. Nevertheless, in *Gpr19* cKO^{Avil} mice, the analgesic effects of adropin in alleviating noxious heat pain were completely lost (Figure 7J). In fact, adropin augmented nocifensive responses to noxious heat in *Gpr19* cKO^{Avil} mice (Figure 7J), suggesting that adropin may be acting on non-Gpr19 targets. This is consistent with the finding that the *in vitro* activity of adropin was only partially lost in *Gpr19* cKO^{Calca} mice (Figures 7C and 7D).

These findings suggest that the analgesic effect of adropin is mediated by Gpr19 in the sensory neurons, while a potential off-target(s) site of action of adropin may be pro-nocifensive.

DISCUSSION

GPCRs are common drug targets. Indeed, over one-third of clinically marketed drugs target GPCRs.[75] The $G_{i/o}$-coupled GPCRs are unique because of their inhibitory action. Besides the well-characterized analgesic $G_{i/o}$-coupled GPCRs, including the opioid receptors, cannabinoid receptors, α2-adrenergic receptors, and metabotropic GABA receptors,[9,10] other $G_{i/o}$-coupled receptors with *in vivo* analgesic efficacy have been recently reported.[76–79] However, few of these studies have defined the exact site of action, and thus the underlying mechanisms remain elusive. Here, we focused on DRG sensory neurons and developed a pipeline for identifying druggable $G_{i/o}$-coupled GPCRs enriched in nociceptors to treat pain. This involved bioinformatically identifying $G_{i/o}$-coupled or orphan GPCRs selectively expressed in CGRP$^+$ sensory neurons, followed by validating expression in human DRGs, evaluating the capacity of agonists/putative agonists to silence DRG neuron excitability *in vitro*, and testing compounds *in vivo* using animal behavior paradigms and conditional receptor loss-of-function alleles to determine the site of action of both desirable and undesirable behavioral consequences of the treatments. With the aid of a mouse DRG neuron genetic toolkit that enables precise manipulation of select sensory neuron subtypes,[5,18,20,80–84] we can identify not only the neuronal subtype(s) responsible for the drug action but also relevant targets within the cell type. Using Htr1b and triptans as an example, conditional loss-of-function experiments revealed Htr1b expressed in CGRP$^+$ sensory neurons, and in particular fast conducting A-fiber nociceptors, as the site of analgesic action of triptans. Htr1b, but not Htr1d, mediates the analgesic effects of triptans, while the adverse effects associated with high doses of triptans are mostly attributed to Htr1b-independent target(s).

Triptans have been primarily used clinically for migraine treatment, and their potential utility in other pain conditions remains largely underexplored. In preclinical models, studies have reported mixed results regarding sumatriptan's analgesic effects in somatic, visceral, or neuropathic pain models.[45–48,85,86] Our findings that triptans exhibit analgesic effects in mouse somatic pain models including acute noxious cold and neuropathic pain are in line with the expression of *Htr1b* in the DRG across all axial levels and suggest a potentially broader therapeutic utility beyond migraine. Although triptans are the first-line treatment for migraine, their mechanisms of action remain unclear.[87,88] At least three modes of action have been proposed: vasoconstriction of vascular smooth muscle, inhibition of trigeminal neurons and peripheral release of neuropeptides including CGRP, and inhibition of central neurotransmitter release in the trigeminocervical complex.[63–65,89] Notably, CGRP inhibitors have recently emerged as a class of migraine therapy.[90] Our findings that *Htr1b* is broadly expressed in CGRP$^+$ sensory neurons in both mouse and human DRGs, triptans silence CGRP$^+$ sensory neurons *in vitro*, sumatriptan decreases glutamate release from sensory neuron synapses in the spinal cord dorsal horn, and that Htr1b functions cell-autonomously in somatosensory neurons to mediate the analgesic properties of triptans support the view that CGRP$^+$ sensory neurons are the primary targets of triptans.

One intriguing finding is the modality-specific analgesic effects of Htr1b agonism, despite its broad expression across most CGRP$^+$ sensory neuron subtypes (Figure 1B). Sumatriptan specifically attenuates noxious cold but not noxious heat or acute mechanical pain (Figures 2A–2E, S4A, and S4B), consistent with previous findings.[48,86] Our functional characterizations of DRG neurons revealed no single DRG neuron subtype exclusively encoding noxious cold.[18,20] Rather, cold responses are distributed across several subtypes, including subsets of Aδ-HTMRs.[18,20] The distributed encoding across multiple subtypes and the broad expression of *Htr1b* among these neurons may account for the efficacy of triptans in noxious cold pain. In contrast, at least six sensory neuron subtypes respond robustly to noxious heat.[18,20] Only two subpopulations express *Htr1b* (Figure 1B). Activating Htr1b in this limited population may be insufficient to block heat-induced nocifensive behaviors. It is unclear why sumatriptan had no effect on noxious mechanical pain in the pinprick assay, given *Htr1b* expression in Aδ-HTMRs and the implication of Aδ-HTMRs in mediating the actions of sumatriptan in mechanical allodynia. Pinprick evokes a strong reflex response. It is possible that moderately inhibiting the relevant sensory neuron populations is insufficient to block this acute, robust behavior. Sumatriptan appeared to be ineffective in inflammatory mechanical and thermal pain (Figures 2F and 2G), consistent with prior findings that intrathecal, but not systemic, application of sumatriptan attenuated inflammatory pain.[48] Interestingly, triptans were more effective in neuropathic pain than in acute pain models (Figures 2H–2M and S4E–S4G), despite a previous report of sumatriptan inefficacy in SNI-induced mechanical allodynia.[48] The basis of this discrepancy is unclear but may reflect differences in SNI surgery (sparing sural vs. tibial nerve). The mechanisms underlying triptan analgesia remain to be determined. We cannot exclude the possibility that Htr1b expression or localization is altered under neuropathic pain states or a potential contribution of changes in the central circuits after nerve injury. Future experiments are needed to address these questions.

Our findings support re-evaluating existing Htr1b agonists for treating nociceptive and neuropathic pain and revisiting Htr1b as a target for analgesic development. Besides Htr1b, we identified several other $G_{i/o}$-coupled GPCRs and orphan receptors potentially $G_{i/o}$-coupled, including Gpr19, whose agonists attenuated excitability of CGRP$^+$ sensory neurons *in vitro*, meriting further investigation with pain behavioral tests and cKO approaches. Indeed, the putative Gpr19 agonist, adropin, exhibited moderate analgesic effects in the noxious heat pain paradigm, despite potential off-target effects. Conditional loss-of-function experiments confirmed that the analgesic effects of adropin rely on Gpr19 in the sensory neurons, while off-target pro-nocifensive effects are mediated by other receptors and/or excitatory signaling pathways. These findings point to Gpr19 as a promising analgesic candidate, and more selective agonists may be therapeutically valuable. Notably, the *Gpr19* cKO mice exhibited hyposensitivity to noxious heat, implicating Gpr19 in noxious

heat detection. Together, our findings highlight the feasibility of targeting GPCRs in DRG sensory neurons to inhibit nociceptors and reveal opportunities for developing pain therapeutics.

Limitations of the study

One limitation of the study is that the identity of sensory neurons whose central inputs were inhibited by sumatriptan in the electrophysiology experiments reported in Figure 3 was not definitively established; *Htr1b* is expressed in both Aδ- and C-fiber CGRP$^+$ neurons, and inputs from both cell types are likely inhibited by sumatriptan. Additionally, the lack of well-defined human DRG neuron subtype marker genes corresponding to physiologically and functionally defined sensory neurons precluded a precise mapping of GPCR expression onto human sensory neuron subtypes, limiting our ability to directly translate the mouse findings to human biology. Our analysis was also restricted to a subset of GPCRs expressed in human DRGs, leaving other potentially relevant GPCRs unexamined. Several interesting orphan GPCRs identified in our screen lack known agonists, limiting their functional characterization and precluding *in vivo* validation of their analgesic potential. Furthermore, other GPCRs expressed in DRG neurons, including G_s- or $G_{q/11}$-coupled receptors, may also hold therapeutic potential but were not explored here. Finally, while our findings support the potential repurposing of triptans for somatic pain conditions beyond migraine, the clinical translation of these findings will require systematic evaluation of triptan efficacy and safety in relevant patient populations.

RESOURCE AVAILABILITY

Lead contact

Further information and requests for resources and reagents should be directed to and will be fulfilled by the lead contact, David Ginty (david_ginty@hms.harvard.edu).

Materials availability

The *Gpr19*^{fl} mouse line is available upon request.

Data and code availability

- All data reported in this study will be shared by the lead contact upon request.
- All original code has been deposited at Zenodo and is publicly available at https://doi.org/10.5281/zenodo.20801945 as of the date of publication.
- Any additional information required to reanalyze the data reported in this work is available from the lead contact upon request.

ACKNOWLEDGMENTS

We thank Dr. Yotam Sagi (Rockefeller University) for the *Htr1b*^{fl} mice, Elke Bentley from the Woolf lab (Boston Children's Hospital) for help with conducting the Hargreaves assay, Dr. Meredith Skiba and Dr. Andrew Kruse (Harvard Medical School) for some of the GPCR-expressing plasmids, Caiying Guo at the HHMI Janelia Research Campus for generating the *Gpr19*^{fl} mouse line, and Ginty lab members for discussions and comments on the manuscript. This work was supported by NIH grants NS097344-05 (D.D.G.), NS132196-02 (D.D.G.), AT011447 (D.D.G.), and NS105076-01 (C.J.W.); the Hock E. Tan and Lisa Yang Center for Autism Research (D.D.G.); the Edward R. and Anne G. Lefler Center for Neurodegenerative Disorders (D.D.G.); and Ono Pharmaceutical. D.D.G. is an investigator of the Howard Hughes Medical Institute (HHMI).

AUTHOR CONTRIBUTIONS

J.P., N.S., and D.D.G. conceived the study. J.P. performed RNAscope on mouse and human DRGs, *in vitro* calcium imaging, *in vitro* cAMP signaling assay, and all animal behavior tests except the paw luminance and Hargreaves assay, with help from B.T.S. and M.M.D. A.M.C. performed the slice electrophysiology experiments. J.Y.X. performed the *in vivo* calcium imaging. X.Z. performed the paw luminance assay and the Hargreaves assay under the supervision of C.J.W. L.Q. and K.L. contributed to the setup of the *in vitro* calcium imaging and the pinprick assay, respectively. J.P. and D.D.G. wrote the paper with input from all authors.

DECLARATION OF INTERESTS

D.D.G. is a scientific founder of Krause Therapeutics and a consultant for Alive Molecular Technologies, Inc. Parts of the work were supported by Ono Pharmaceutical. A patent application related to this work titled "Targeting $G_{i/o}$-coupled GPCRs to treat pain" has been submitted.

STAR★METHODS

Detailed methods are provided in the online version of this paper and include the following:

- KEY RESOURCES TABLE
- EXPERIMENTAL MODEL AND STUDY PARTICIPANT DETAILS
- METHOD DETAILS
  - Drug treatments
  - *In situ* hybridization (RNAscope)
  - *In vitro* cAMP signaling assay
  - DRG neuron dissociation and culture
  - *In vitro* calcium imaging
  - *In vivo* calcium imaging
  - Spared nerve injury (SNI)
  - Open field
  - Cold and hot plate
  - von Frey (VF)
  - Dynamic brush
  - Pinprick
  - Inflammatory pain (zymosan model)
  - Temperature preference
  - Guarding behavior
  - Paw luminance assay
  - Texture preference (sandpaper)
  - Novel object test
  - Balance beam
  - Electrocardiogram
  - Slice electrophysiology
- QUANTIFICATION AND STATISTICAL ANALYSIS

SUPPLEMENTAL INFORMATION

Supplemental information can be found online at https://doi.org/10.1016/j.xcrm.2026.102949.

Received: February 9, 2026
Revised: April 28, 2026
Accepted: July 3, 2026
Published: July 30, 2026

REFERENCES

1. Raja, S.N., Carr, D.B., Cohen, M., Finnerup, N.B., Flor, H., Gibson, S., Keefe, F.J., Mogil, J.S., Ringkamp, M., Sluka, K.A., et al. (2020). The revised International Association for the Study of Pain definition of pain: concepts, challenges, and compromises. Pain *161*, 1976–1982. https://doi.org/10.1097/j.pain.0000000000001939.

2. Basbaum, A.I., Bautista, D.M., Scherrer, G., and Julius, D. (2009). Cellular and molecular mechanisms of pain. Cell *139*, 267–284. https://doi.org/10.1016/j.cell.2009.09.028.
3. Dong, X., Han, S., Zylka, M.J., Simon, M.I., and Anderson, D.J. (2001). A diverse family of GPCRs expressed in specific subsets of nociceptive sensory neurons. Cell *106*, 619–632. https://doi.org/10.1016/s0092-8674(01)00483-4.
4. Usoskin, D., Furlan, A., Islam, S., Abdo, H., Lönnerberg, P., Lou, D., Hjerling-Leffler, J., Haeggström, J., Kharchenko, O., Kharchenko, P.V., et al. (2015). Unbiased classification of sensory neuron types by large-scale single-cell RNA sequencing. Nat. Neurosci. *18*, 145–153. https://doi.org/10.1038/nn.3881.
5. Sharma, N., Flaherty, K., Lezgiyeva, K., Wagner, D.E., Klein, A.M., and Ginty, D.D. (2020). The emergence of transcriptional identity in somatosensory neurons. Nature *577*, 392–398. https://doi.org/10.1038/s41586-019-1900-1.
6. Pierce, K.L., Premont, R.T., and Lefkowitz, R.J. (2002). Seven-transmembrane receptors. Nat. Rev. Mol. Cell Biol. *3*, 639–650. https://doi.org/10.1038/nrm908.
7. Salzer, I., Ray, S., Schicker, K., and Boehm, S. (2019). Nociceptor Signalling through ion Channel Regulation via GPCRs. Int. J. Mol. Sci. *20*, 2488. https://doi.org/10.3390/ijms20102488.
8. Geppetti, P., Veldhuis, N.A., Lieu, T., and Bunnett, N.W. (2015). G Protein-Coupled Receptors: Dynamic Machines for Signaling Pain and Itch. Neuron *88*, 635–649. https://doi.org/10.1016/j.neuron.2015.11.001.
9. Pan, H.L., Wu, Z.Z., Zhou, H.Y., Chen, S.R., Zhang, H.M., and Li, D.P. (2008). Modulation of pain transmission by G-protein-coupled receptors. Pharmacol. Ther. *117*, 141–161. https://doi.org/10.1016/j.pharmthera.2007.09.003.
10. Stone, L.S., and Molliver, D.C. (2009). In search of analgesia: emerging roles of GPCRs in pain. Mol. Interv. *9*, 234–251. https://doi.org/10.1124/mi.9.5.7.
11. Yudin, Y., and Rohacs, T. (2018). Inhibitory G(i/O)-coupled receptors in somatosensory neurons: Potential therapeutic targets for novel analgesics. Mol. Pain *14*, 1744806918763646. https://doi.org/10.1177/1744806918763646.
12. Kang, H.J., Krumm, B.E., Tassou, A., Geron, M., DiBerto, J.F., Kapolka, N.J., Gumpper, R.H., Sakamoto, K., Dewran Kocak, D., Olsen, R.H., et al. (2024). Structure-guided design of a peripherally restricted chemogenetic system. Cell *187*, 7433–7449.e20. https://doi.org/10.1016/j.cell.2024.11.001.
13. Günther, T., Dasgupta, P., Mann, A., Miess, E., Kliewer, A., Fritzwanker, S., Steinborn, R., and Schulz, S. (2018). Targeting multiple opioid receptors - improved analgesics with reduced side effects? Br. J. Pharmacol. *175*, 2857–2868. https://doi.org/10.1111/bph.13809.
14. Johnston, M.M., and Rapoport, A.M. (2010). Triptans for the management of migraine. Drugs *70*, 1505–1518. https://doi.org/10.2165/11537990-000000000-00000.
15. Pöpping, D.M., Elia, N., Marret, E., Wenk, M., and Tramèr, M.R. (2009). Clonidine as an adjuvant to local anesthetics for peripheral nerve and plexus blocks: a meta-analysis of randomized trials. Anesthesiology *111*, 406–415. https://doi.org/10.1097/ALN.0b013e3181aae897.
16. Hussain, N., Grzywacz, V.P., Ferreri, C.A., Atrey, A., Banfield, L., Shaparin, N., and Vydyanathan, A. (2017). Investigating the Efficacy of Dexmedetomidine as an Adjuvant to Local Anesthesia in Brachial Plexus Block: A Systematic Review and Meta-Analysis of 18 Randomized Controlled Trials. Reg. Anesth. Pain Med. *42*, 184–196. https://doi.org/10.1097/AAP.0000000000000564.
17. Zeisel, A., Hochgerner, H., Lönnerberg, P., Johnsson, A., Memic, F., van der Zwan, J., Häring, M., Braun, E., Borm, L.E., La Manno, G., et al. (2018). Molecular Architecture of the Mouse Nervous System. Cell *174*, 999–1014.e22. https://doi.org/10.1016/j.cell.2018.06.021.
18. Qi, L., et al. (2024). mouse DRG genetic toolkit reveals morphological and physiological diversity of somatosensory neuron subtypes. Cell *187*, 1508. https://doi.org/10.1016/j.cell.2024.02.006.
19. Zheng, Y., Liu, P., Bai, L., Trimmer, J.S., Bean, B.P., and Ginty, D.D. (2019). Deep Sequencing of Somatosensory Neurons Reveals Molecular Determinants of Intrinsic Physiological Properties. Neuron *103*, 598–616.e7. https://doi.org/10.1016/j.neuron.2019.05.039.
20. Lezgiyeva, K., Liu, J., Nguyen, K., DeLisle, M.M., Ko, F.C., Fullam, S., Obeidat, A.M., Turecek, J., Alkislar, I., Lehnert, B.P., et al. (2026). Fast-conducting mechanonociceptors uniquely engage reflexive and affective pain circuitry to drive protective responses. Neuron. https://doi.org/10.1016/j.neuron.2026.06.014.
21. Poll, B.G., Chen, L., Chou, C.L., Raghuram, V., and Knepper, M.A. (2021). Landscape of GPCR expression along the mouse nephron. Am. J. Physiol. Renal Physiol. *321*, F50–F68. https://doi.org/10.1152/ajprenal.00077.2021.
22. Rostock, C., Schrenk-Siemens, K., Pohle, J., and Siemens, J. (2018). Human vs. Mouse Nociceptors - Similarities and Differences. Neuroscience *387*, 13–27. https://doi.org/10.1016/j.neuroscience.2017.11.047.
23. Tavares-Ferreira, D., Shiers, S., Ray, P.R., Wangzhou, A., Jeevakumar, V., Sankaranarayanan, I., Cervantes, A.M., Reese, J.C., Chamessian, A., Copits, B.A., et al. (2022). Spatial transcriptomics of dorsal root ganglia identifies molecular signatures of human nociceptors. Sci. Transl. Med. *14*, eabj8186. https://doi.org/10.1126/scitranslmed.abj8186.
24. Shiers, S., Klein, R.M., and Price, T.J. (2020). Quantitative differences in neuronal subpopulations between mouse and human dorsal root ganglia demonstrated with RNAscope in situ hybridization. Pain *161*, 2410–2424. https://doi.org/10.1097/j.pain.0000000000001973.
25. Bhuiyan, S.A. (2025). A Reference Atlas of the Human Dorsal Root Ganglion. Preprint at bioRxiv. https://doi.org/10.1101/2025.11.05.686654.
26. Nguyen, M.Q., von Buchholtz, L.J., Reker, A.N., Ryba, N.J., and Davidson, S. (2021). Single-nucleus transcriptomic analysis of human dorsal root ganglion neurons. eLife *10*, e71752. https://doi.org/10.7554/eLife.71752.
27. Yu, H., Nagi, S.S., Usoskin, D., Hu, Y., Kupari, J., Bouchatta, O., Yan, H., Cranfill, S.L., Gautam, M., Su, Y., et al. (2024). Leveraging deep single-soma RNA sequencing to explore the neural basis of human somatosensation. Nat. Neurosci. *27*, 2326–2340. https://doi.org/10.1038/s41593-024-01794-1.
28. Jasper, J.R. (1998). Ligand efficacy and potency at recombinant alpha2 adrenergic receptors: agonist-mediated [35S]GTPgammaS binding. Biochem. Pharmacol. *55*, 1035–1043.
29. Proudman, R.G.W., Akinaga, J., and Baker, J.G. (2022). The signaling and selectivity of alpha-adrenoceptor agonists for the human alpha2A, alpha2B and alpha2C-adrenoceptors and comparison with human alpha1 and beta-adrenoceptors. Pharmacol. Res. Perspect. *10*, e01003. https://doi.org/10.1002/prp2.1003.
30. Rao, A., and Herr, D.R. (2017). G protein-coupled receptor GPR19 regulates E-cadherin expression and invasion of breast cancer cells. Biochim. Biophys. Acta. Mol. Cell Res. *1864*, 1318–1327. https://doi.org/10.1016/j.bbamcr.2017.05.001.
31. Stein, L.M., Yosten, G.L.C., and Samson, W.K. (2016). Adropin acts in brain to inhibit water drinking: potential interaction with the orphan G protein-coupled receptor, GPR19. Am. J. Physiol. Regul. Integr. Comp. Physiol. *310*, R476–R480. https://doi.org/10.1152/ajpregu.00511.2015.
32. Wang, J., Simonavicius, N., Wu, X., Swaminath, G., Reagan, J., Tian, H., and Ling, L. (2006). Kynurenic acid as a ligand for orphan G protein-coupled receptor GPR35. J. Biol. Chem. *281*, 22021–22028. https://doi.org/10.1074/jbc.M603503200.
33. Southern, C., Cook, J.M., Neetoo-Isseljee, Z., Taylor, D.L., Kettleborough, C.A., Merritt, A., Bassoni, D.L., Raab, W.J., Quinn, E., Wehrman, T.S., et al. (2013). Screening beta-arrestin recruitment for the identification of natural ligands for orphan G-protein-coupled receptors. SLAS Discovery *18*, 599–609. https://doi.org/10.1177/1087057113475480.

34. Taniguchi, Y., Tonai-Kachi, H., and Shinjo, K. (2006). Zaprinast, a well-known cyclic guanosine monophosphate-specific phosphodiesterase inhibitor, is an agonist for GPR35. FEBS Lett. *580*, 5003–5008. https://doi.org/10.1016/j.febslet.2006.08.015.

35. Felsing, D.E., Raval, S., Jain, M., Xi, S., and Allen, J.A. (2019). Experimental Biology 2019 Meeting, *667.668* (The FASEB Journal)).

36. Sanematsu, K., Yoshida, R., Shigemura, N., and Ninomiya, Y. (2014). Structure, function, and signaling of taste G-protein-coupled receptors. Curr. Pharm. Biotechnol. *15*, 951–961. https://doi.org/10.2174/1389201015666140922105911.

37. Behan, D.P., and Chalmers, D.T. (2001). The use of constitutively active receptors for drug discovery at the G protein-coupled receptor gene pool. Curr. Opin. Drug Discov. Devel. *4*, 548–560.

38. Chen, G., Way, J., Armour, S., Watson, C., Queen, K., Jayawickreme, C.K., Chen, W.J., and Kenakin, T. (2000). Use of constitutive G protein-coupled receptor activity for drug discovery. Mol. Pharmacol. *57*, 125–134.

39. Ngo, T., Coleman, J.L., and Smith, N.J. (2015). Using constitutive activity to define appropriate high-throughput screening assays for orphan g protein-coupled receptors. Methods Mol. Biol. *1272*, 91–106. https://doi.org/10.1007/978-1-4939-2336-6_7.

40. Bond, R.A., and Ijzerman, A.P. (2006). Recent developments in constitutive receptor activity and inverse agonism, and their potential for GPCR drug discovery. Trends Pharmacol. Sci. *27*, 92–96. https://doi.org/10.1016/j.tips.2005.12.007.

41. Bresnick, J.N., Skynner, H.A., Chapman, K.L., Jack, A.D., Zamiara, E., Negulescu, P., Beaumont, K., Patel, S., and McAllister, G. (2003). Identification of signal transduction pathways used by orphan g protein-coupled receptors. Assay Drug Dev. Technol. *1*, 239–249. https://doi.org/10.1089/15406580360545053.

42. Teichert, R.W., Smith, N.J., Raghuraman, S., Yoshikami, D., Light, A.R., and Olivera, B.M. (2012). Functional profiling of neurons through cellular neuropharmacology. Proc. Natl. Acad. Sci. USA *109*, 1388–1395. https://doi.org/10.1073/pnas.1118833109.

43. Song, H., Yao, E., Lin, C., Gacayan, R., Chen, M.H., and Chuang, P.T. (2012). Functional characterization of pulmonary neuroendocrine cells in lung development, injury, and tumorigenesis. Proc. Natl. Acad. Sci. USA *109*, 17531–17536. https://doi.org/10.1073/pnas.1207238109.

44. Subcutaneous Sumatriptan International Study Group (1991). Treatment of migraine attacks with sumatriptan. N. Engl. J. Med. *325*, 316–321. https://doi.org/10.1056/NEJM199108013250504.

45. Afshari, K., Dehdashtian, A., Haddad, N.S., Jazaeri, S.Z., Ursu, D.C., Khalilzadeh, M., Haj-Mirzaian, A., Shakiba, S., Burns, T.C., Tavangar, S.M., et al. (2021). Sumatriptan improves the locomotor activity and neuropathic pain by modulating neuroinflammation in rat model of spinal cord injury. Neurol. Res. *43*, 29–39. https://doi.org/10.1080/01616412.2020.1819090.

46. Khalilzadeh, M., Panahi, G., Rashidian, A., Hadian, M.R., Abdollahi, A., Afshari, K., Shakiba, S., Norouzi-Javidan, A., Rahimi, N., Momeny, M., and Dehpour, A.R. (2018). The protective effects of sumatriptan on vincristine - induced peripheral neuropathy in a rat model. Neurotoxicology *67*, 279–286. https://doi.org/10.1016/j.neuro.2018.06.012.

47. Kayser, V., Aubel, B., Hamon, M., and Bourgoin, S. (2002). The antimigraine 5-HT 1B/1D receptor agonists, sumatriptan, zolmitriptan and dihydroergotamine, attenuate pain-related behaviour in a rat model of trigeminal neuropathic pain. Br. J. Pharmacol. *137*, 1287–1297. https://doi.org/10.1038/sj.bjp.0704979.

48. Nikai, T., Basbaum, A.I., and Ahn, A.H. (2008). Profound reduction of somatic and visceral pain in mice by intrathecal administration of the anti-migraine drug, sumatriptan. Pain *139*, 533–540. https://doi.org/10.1016/j.pain.2008.06.002.

49. Shields, S.D., Eckert, W.A., 3rd, and Basbaum, A.I. (2003). Spared nerve injury model of neuropathic pain in the mouse: a behavioral and anatomic analysis. J. Pain *4*, 465–470. https://doi.org/10.1067/s1526-5900(03)00781-8.

50. Decosterd, I., and Woolf, C.J. (2000). Spared nerve injury: an animal model of persistent peripheral neuropathic pain. Pain *87*, 149–158. https://doi.org/10.1016/S0304-3959(00)00276-1.

51. Mathieson, S., Lin, C.W.C., Underwood, M., and Eldabe, S. (2020). Pregabalin and gabapentin for pain. BMJ *369*, m1315. https://doi.org/10.1136/bmj.m1315.

52. Zhang, Z., Roberson, D.P., Kotoda, M., Boivin, B., Bohnslav, J.P., González-Cano, R., Yarmolinsky, D.A., Turnes, B.L., Wimalasena, N.K., Neufeld, S.Q., et al. (2022). Automated preclinical detection of mechanical pain hypersensitivity and analgesia. Pain *163*, 2326–2336. https://doi.org/10.1097/j.pain.0000000000002680.

53. Peroutka, S.J., and McCarthy, B.G. (1989). Sumatriptan (GR 43175) interacts selectively with 5-HT1B and 5-HT1D binding sites. Eur. J. Pharmacol. *163*, 133–136. https://doi.org/10.1016/0014-2999(89)90406-8.

54. Comer, M.B. (2002). Pharmacology of the selective 5-HT(1B/1D) agonist frovatriptan. Headache *42*, S47–S53. https://doi.org/10.1046/j.1526-4610.42.s2.2.x.

55. Napier, C., Stewart, M., Melrose, H., Hopkins, B., McHarg, A., and Wallis, R. (1999). Characterisation of the 5-HT receptor binding profile of eletriptan and kinetics of [3H]eletriptan binding at human 5-HT1B and 5-HT1D receptors. Eur. J. Pharmacol. *368*, 259–268.

56. Virk, M.S., Sagi, Y., Medrihan, L., Leung, J., Kaplitt, M.G., and Greengard, P. (2016). Opposing roles for serotonin in cholinergic neurons of the ventral and dorsal striatum. Proc. Natl. Acad. Sci. USA *113*, 734–739. https://doi.org/10.1073/pnas.1524183113.

57. Tfelt-Hansen, P.C. (2010). Does sumatriptan cross the blood-brain barrier in animals and man? J. Headache Pain *11*, 5–12. https://doi.org/10.1007/s10194-009-0170-y.

58. Svane, N., Bällgren, F., Ginosyan, A., Kristensen, M., Brodin, B., and Loryan, I. (2024). Regional distribution of unbound eletriptan and sumatriptan in the CNS and PNS in rats: implications for a potential central action. J. Headache Pain *25*, 187. https://doi.org/10.1186/s10194-024-01894-0.

59. Messina, R., Christensen, R.H., Cetta, I., Ashina, M., and Filippi, M. (2023). Imaging the brain and vascular reactions to headache treatments: a systematic review. J. Headache Pain *24*, 58. https://doi.org/10.1186/s10194-023-01590-5.

60. Muzzi, M., Zecchi, R., Ranieri, G., Urru, M., Tofani, L., De Cesaris, F., Panconesi, A., and Chiarugi, A. (2020). Ultra-rapid brain uptake of subcutaneous sumatriptan in the rat: Implication for cluster headache treatment. Cephalalgia *40*, 330–336. https://doi.org/10.1177/0333102419896370.

61. Stirling, L.C., Forlani, G., Baker, M.D., Wood, J.N., Matthews, E.A., Dickenson, A.H., and Nassar, M.A. (2005). Nociceptor-specific gene deletion using heterozygous NaV1.8-Cre recombinase mice. Pain *113*, 27–36. https://doi.org/10.1016/j.pain.2004.08.015.

62. Nassar, M.A., Stirling, L.C., Forlani, G., Baker, M.D., Matthews, E.A., Dickenson, A.H., and Wood, J.N. (2004). Nociceptor-specific gene deletion reveals a major role for Nav1.7 (PN1) in acute and inflammatory pain. Proc. Natl. Acad. Sci. USA *101*, 12706–12711. https://doi.org/10.1073/pnas.0404915101.

63. Storer, R.J., and Goadsby, P.J. (1997). Microiontophoretic application of serotonin (5HT)1B/1D agonists inhibits trigeminal cell firing in the cat. Brain *120*, 2171–2177. https://doi.org/10.1093/brain/120.12.2171.

64. Choi, I.S., Cho, J., An, C., Jung, J., Hur, Y., Choi, J., and Jang, I. (2012). 5-HT(1B) receptors inhibit glutamate release from primary afferent terminals in rat medullary dorsal horn neurons. Br. J. Pharmacol. *167*, 356–367. https://doi.org/10.1111/j.1476-5381.2012.01964.x.

65. Levy, D., Jakubowski, M., and Burstein, R. (2004). Disruption of communication between peripheral and central trigeminovascular neurons mediates the antimigraine action of 5HT 1B/1D receptor agonists. Proc. Natl. Acad. Sci. USA *101*, 4274–4279. https://doi.org/10.1073/pnas.0306147101.

66. Lau, J., Minett, M.S., Zhao, J., Dennehy, U., Wang, F., Wood, J.N., and Bogdanov, Y.D. (2011). Temporal control of gene deletion in sensory

ganglia using a tamoxifen-inducible Advillin-Cre-ERT2 recombinase mouse. Mol. Pain *7*, 100. https://doi.org/10.1186/1744-8069-7-100.

67. Choi, S., Hachisuka, J., Brett, M.A., Magee, A.R., Omori, Y., Iqbal, N.U.A., Zhang, D., DeLisle, M.M., Wolfson, R.L., Bai, L., et al. (2020). Parallel ascending spinal pathways for affective touch and pain. Nature *587*, 258–263. https://doi.org/10.1038/s41586-020-2860-1.

68. Visser, W.H., Jaspers, N.M., de Vriend, R.H., and Ferrari, M.D. (1996). Chest symptoms after sumatriptan: a two-year clinical practice review in 735 consecutive migraine patients. Cephalalgia *16*, 554–559. https://doi.org/10.1046/j.1468-2982.1996.1608554.x.

69. Ottervanger, J.P., Valkenburg, H.A., Grobbee, D.E., and Stricker, B.H. (1997). Characteristics and determinants of sumatriptan-associated chest pain. Arch. Neurol. *54*, 1387–1392. https://doi.org/10.1001/archneur.1997.00550230054017.

70. Liu, W.H., Hu, H.M., Li, C., Shi, Q., Liu, C.H., Liu, A.X., Li, Y.F., Zhang, Y., Mao, P., and Fan, B.F. (2024). Real-world study of adverse events associated with triptan use in migraine treatment based on the U.S. Food and Drug Administration (FDA) adverse event reporting system (FAERS) database. J. Headache Pain *25*, 206. https://doi.org/10.1186/s10194-024-01913-0.

71. Humphrey, P.P., Feniuk, W., Marriott, A.S., Tanner, R.J., Jackson, M.R., and Tucker, M.L. (1991). Preclinical studies on the anti-migraine drug, sumatriptan. Eur. Neurol. *31*, 282–290. https://doi.org/10.1159/000116755.

72. Perren, M.J., Feniuk, W., and Humphrey, P.P. (1989). The selective closure of feline carotid arteriovenous anastomoses (AVAs) by GR43175. Cephalalgia *9*, 41–46. https://doi.org/10.1111/J.1468-2982.1989.TB00071.X.

73. Lakso, M., Pichel, J.G., Gorman, J.R., Sauer, B., Okamoto, Y., Lee, E., Alt, F.W., and Westphal, H. (1996). Efficient in vivo manipulation of mouse genomic sequences at the zygote stage. Proc. Natl. Acad. Sci. USA *93*, 5860–5865. https://doi.org/10.1073/pnas.93.12.5860.

74. Martin, G.R. (1997). Pre-clinical pharmacology of zolmitriptan (Zomig; formerly 311C90), a centrally and peripherally acting 5HT1B/1D agonist for migraine. Cephalalgia *17*, 4–14. https://doi.org/10.1177/0333102497017S1802.

75. Sriram, K., and Insel, P.A. (2018). G Protein-Coupled Receptors as Targets for Approved Drugs: How Many Targets and How Many Drugs? Mol. Pharmacol. *93*, 251–258. https://doi.org/10.1124/mol.117.111062.

76. Durante, M., Squillace, S., Lauro, F., Giancotti, L.A., Coppi, E., Cherchi, F., Di Cesare Mannelli, L., Ghelardini, C., Kolar, G., Wahlman, C., et al. (2021). Adenosine A3 agonists reverse neuropathic pain via T cell-mediated production of IL-10. J. Clin. Investig. *131*, e139299. https://doi.org/10.1172/JCI139299.

77. Chen, Z., Janes, K., Chen, C., Doyle, T., Bryant, L., Tosh, D.K., Jacobson, K.A., and Salvemini, D. (2012). Controlling murine and rat chronic pain through A3 adenosine receptor activation. FASEB J. *26*, 1855–1865. https://doi.org/10.1096/fj.11-201541.

78. Cho, P.S., Lee, H.K., Choi, Y.I., Choi, S.I., Lim, J.Y., Kim, M., Kim, H., Jung, S.J., and Hwang, S.W. (2021). GPR171 Activation Modulates Nociceptor Functions, Alleviating Pathologic Pain. Biomedicines *9*, 256. https://doi.org/10.3390/biomedicines9030256.

79. Ram, A., Edwards, T., McCarty, A., Afrose, L., McDermott, M.V., and Bobeck, E.N. (2021). GPR171 Agonist Reduces Chronic Neuropathic and Inflammatory Pain in Male, But Not Female Mice. Front. Pain Res. *2*, 695396. https://doi.org/10.3389/fpain.2021.695396.

80. Luo, W., Enomoto, H., Rice, F.L., Milbrandt, J., and Ginty, D.D. (2009). Molecular identification of rapidly adapting mechanoreceptors and their developmental dependence on ret signaling. Neuron *64*, 841–856. https://doi.org/10.1016/j.neuron.2009.11.003.

81. Li, L., Rutlin, M., Abraira, V.E., Cassidy, C., Kus, L., Gong, S., Jankowski, M.P., Luo, W., Heintz, N., Koerber, H.R., et al. (2011). The functional organization of cutaneous low-threshold mechanosensory neurons. Cell *147*, 1615–1627. https://doi.org/10.1016/j.cell.2011.11.027.

82. Rutlin, M., Ho, C.Y., Abraira, V.E., Cassidy, C., Bai, L., Woodbury, C.J., and Ginty, D.D. (2014). The cellular and molecular basis of direction selectivity of Adelta-LTMRs. Cell *159*, 1640–1651. https://doi.org/10.1016/j.cell.2014.11.038.

83. Bai, L., Lehnert, B.P., Liu, J., Neubarth, N.L., Dickendesher, T.L., Nwe, P.H., Cassidy, C., Woodbury, C.J., and Ginty, D.D. (2015). Genetic Identification of an Expansive Mechanoreceptor Sensitive to Skin Stroking. Cell *163*, 1783–1795. https://doi.org/10.1016/j.cell.2015.11.060.

84. Abraira, V.E., Kuehn, E.D., Chirila, A.M., Springel, M.W., Toliver, A.A., Zimmerman, A.L., Orefice, L.L., Boyle, K.A., Bai, L., Song, B.J., et al. (2017). The Cellular and Synaptic Architecture of the Mechanosensory Dorsal Horn. Cell *168*, 295–310.e19. https://doi.org/10.1016/j.cell.2016.12.010.

85. Vera-Portocarrero, L.P., Ossipov, M.H., King, T., and Porreca, F. (2008). Reversal of inflammatory and noninflammatory visceral pain by central or peripheral actions of sumatriptan. Gastroenterology *135*, 1369–1378. https://doi.org/10.1053/j.gastro.2008.06.085.

86. Ottani, A., Ferraris, E., Giuliani, D., Mioni, C., Bertolini, A., Sternieri, E., and Ferrari, A. (2004). Effect of sumatriptan in different models of pain in rats. Eur. J. Pharmacol. *497*, 181–186. https://doi.org/10.1016/j.ejphar.2004.06.053.

87. Ahn, A.H., and Basbaum, A.I. (2005). Where do triptans act in the treatment of migraine? Pain *115*, 1–4. https://doi.org/10.1016/j.pain.2005.03.008.

88. Goadsby, P.J., Holland, P.R., Martins-Oliveira, M., Hoffmann, J., Schankin, C., and Akerman, S. (2017). Pathophysiology of Migraine: A Disorder of Sensory Processing. Physiol. Rev. *97*, 553–622. https://doi.org/10.1152/physrev.00034.2015.

89. Goadsby, P.J. (1998). Serotonin receptors and the acute attack of migraine. Clin. Neurosci. *5*, 18–23.

90. Mohanty, D., and Lippmann, S. (2020). CGRP Inhibitors for Migraine. Innov. Clin. Neurosci. *17*, 39–40.

91. Rodríguez, C.I., Buchholz, F., Galloway, J., Sequerra, R., Kasper, J., Ayala, R., Stewart, A.F., and Dymecki, S.M. (2000). High-efficiency deleter mice show that FLPe is an alternative to Cre-loxP. Nat. Genet. *25*, 139–140. https://doi.org/10.1038/75973.

92. Dubbs, A., Guevara, J., and Yuste, R. (2016). Fast Motion Correction for Calcium Imaging. Front. Neuroinform. *10*, 6. https://doi.org/10.3389/fninf.2016.00006.

93. Huey, E.L., Turecek, J., Delisle, M.M., Mazor, O., Romero, G.E., Dua, M., Sarafis, Z.K., Hobble, A., Booth, K.T., Goodrich, L.V., et al. (2025). The auditory midbrain mediates tactile vibration sensing. Cell *188*, 104–120.e18. https://doi.org/10.1016/j.cell.2024.11.014.

94. Duan, B., Cheng, L., Bourane, S., Britz, O., Padilla, C., Garcia-Campmany, L., Krashes, M., Knowlton, W., Velasquez, T., Ren, X., et al. (2014). Identification of spinal circuits transmitting and gating mechanical pain. Cell *159*, 1417–1432. https://doi.org/10.1016/j.cell.2014.11.003.

95. Meller, S.T., and Gebhart, G.F. (1997). Intraplantar zymosan as a reliable, quantifiable model of thermal and mechanical hyperalgesia in the rat. Eur. J. Pain *1*, 43–52. https://doi.org/10.1016/s1090-3801(97)90052-5.

96. Hakim, S., Jain, A., Adamson, S.S., Petrova, V., Indajang, J., Kim, H.W., Kawaguchi, R., Wang, Q., Duran, E.S., Nelson, D., et al. (2025). Macrophages protect against sensory axon loss in peripheral neuropathy. Nature *640*, 212–220. https://doi.org/10.1038/s41586-024-08535-1.

97. Ting, J.T., Daigle, T.L., Chen, Q., and Feng, G. (2014). Acute brain slice methods for adult and aging animals: application of targeted patch clamp analysis and optogenetics. Methods Mol. Biol. *1183*, 221–242. https://doi.org/10.1007/978-1-4939-1096-0_14.

98. Zhang, D., Turecek, J., Choi, S., Delisle, M., Pamplona, C.L., Meltzer, S., and Ginty, D.D. (2024). C-LTMRs evoke wet dog shakes via the spinoparabrachial pathway. Science *386*, 686–692. https://doi.org/10.1126/science.adq8834.

STAR★METHODS

KEY RESOURCES TABLE

REAGENT or RESOURCE	SOURCE	IDENTIFIER
Biological samples		
Human DRG tissue (L5)	AnaBios	N/A
Chemicals, peptides, and recombinant proteins		
Tamoxifen	Sigma-Aldrich	Cat#T5648-1g
Sunflower seed oil	Sigma-Aldrich	Cat#S5007
Isoflurane solution	Henry Schein	Cat#029405
Paraformaldehyde 32% Aqueous Solution EM Grade	Electron Microscopy Sciences	Cat#15714-S
Sumatriptan Succinate	Sigma-Aldrich	Cat#PHR2580-500MG
Zolmitriptan	Sigma-Aldrich	Cat#SML0248-10MG
Dexmedetomidine hydrochloride	Sigma-Aldrich	Cat#SML0956-10MG
Somatostatin	Sigma-Aldrich	Cat#S9129-1MG
Zaprinast	Sigma-Aldrich	Cat#Z0878-25MG
Ammonium pyrrolidinedithiocarbamate (PDTC)	Sigma-Aldrich	Cat#P8765-1G
Zymosan A from Saccharomyces cerevisiae	Sigma-Aldrich	Cat#Z4250
Gabapentin	Sigma-Aldrich	Cat#PHR1049-1G
(−)-Isoproterenol hydrochloride	Sigma-Aldrich	Cat#I6504-1G
Kynurenic acid	Sigma-Aldrich	Cat#K3375-250MG
Neg-50 Frozen Section Medium	VWR	Cat#21008–918
DAPI Fluoromount-G	SouthernBiotech	Cat#0100-20
Adropin (34–76) (human, mouse, rat)	TargetMol	Cat#T76664
Critical commercial assays		
RNAscope Multiplex Fluorescent V2 Assay	ACDBio	Cat#322381, Cat#323110
GloSensor cAMP assay	Promega	Cat#E1290
Experimental models: Cell lines		
Cell line: HEK-293T	Sigma-Aldrich	Cat#12022001-1VL
Experimental models: Organisms/strains		
Mouse: $Gpr19^{fl}$	This paper	N/A
Mouse: wt: C57Bl/6J	Jackson Laboratory	RRID:IMSR_JAX:000664
Mouse: $Avil^{CreER}$: Tg(Avil-icre/ERT2)AJwo/J	Jackson Laboratory, Lau et al.[66]	RRID:IMSR_JAX:032027
Mouse: $Calca^{CreER}$	Song et al.[43]	N/A
Mouse: $Bmpr1b^{Cre}$	Sharma et al.[5]	RRID:IMSR_JAX:039561
Mouse: $Smr2^{Cre}$: $Smr2^{tm1(cre)Ddg}$/J	Jackson Laboratory, Qi et al.[18]	RRID:IMSR_JAX:039562
Mouse: $Na_v1.8^{Cre}$: B6.129(Cg)-$Scn10a^{tm2(cre)Jwo}$/TjpJ	Jackson Laboratory, Nassar et al.[62]	RRID:IMSR_JAX:036564
Mouse: $EIIa^{Cre}$: B6.FVB-Tg(EIIa-cre)C5379Lmgd/J	Jackson Laboratory, Lakso et al.[73]	RRID:IMSR_JAX:003724
Mouse: $Htr1b^{fl}$	Virk et al.[56]	N/A
Mouse: $Calca^{FlpE}$	Choi et al.[67]	N/A
Mouse: $Actb^{FlpE}$: B6.Cg-Tg(ACTFLPe)9205Dym/J	Jackson Laboratory, Rodriguez et al.[91]	RRID:IMSR_JAX:005703
Mouse: Ai148: B6.Cg-$Igs7^{tm148.1(tetO-GCaMP6f,CAG-tTA2)Hze}$/J	Jackson Laboratory	RRID:IMSR_JAX:030328
Mouse: Ai95: B6J.Cg-$Gt(ROSA)26Sor^{tm95.1(CAG-GCaMP6f)Hze}$/MwarJ	Jackson Laboratory	RRID:IMSR_JAX:028865
Mouse: Ai32: B6.Cg-$Gt(ROSA)26Sor^{tm32(CAG-COP4*H134R/EYFP)Hze}$/J	Jackson Laboratory	RRID:IMSR_JAX:024109
Mouse: Ai195: B6;129S6-$Igs7^{tm195(tetO-GCaMP7s,CAG-tTA2)Tasic}$/J	Jackson Laboratory	RRID:IMSR_JAX:034112

(Continued on next page)

Continued

REAGENT or RESOURCE	SOURCE	IDENTIFIER
Recombinant DNA		
pcDNA3.1^{+}-HA-FLAG-GPR19	This paper	N/A
pcDNA3.1^{+}-HA-FLAG-GPR35	This paper	N/A
pcDNA3.1^{+}-HA-FLAG-GPR149	This paper	N/A
pcDNA3.1^{+}/C-(K)DYK-ADRA2A	GenScript	Cat#OHu19050
pcDNA3-FLAG-ADRB2	Addgene	Cat#14697
pGloSensor-22F cAMP plasmid	Promega	Cat#E2301
Software and algorithms		
MATLAB	Mathworks	https://www.mathworks.com/products/MATLAB.html; RRID: SCR_001622
Custom MATLAB code for the pinprick assay	This paper	Zenodo: https://doi.org/10.5281/zenodo.20801945
ImageJ	NIH	https://ImageJ.nih.gov/ij/
Arduino IDE	Arduino	https://docs.arduino.cc/software/ide/; RRID: SCR_024884
Pylon Viewer Software	Basler	https://www.baslerweb.com/en-us/software/pylon/pylon-viewer/
Clampfit software	Molecular Devices	https://www.moleculardevices.com/products/axon-patch-clamp-system/acquisition-and-analysis-software/pclamp-software-suite
EzCG Signal Analyses Software	Mouse Specifics	https://mousespecifics.com/ezcg-software/

EXPERIMENTAL MODEL AND STUDY PARTICIPANT DETAILS

Mice were used as the animal model and human DRG tissue from donors was used to assess the conservation of expression. All animal-related procedures were approved by the Harvard Medical School Institutional Animal Care and Use Committees (IACUC) and complied with the Guide for Animal Care and Use of Laboratory Animals. Mice were group housed in standard housing on a 12 h light/12 h dark cycle with free access to food and water. Male and female mice of mixed genetic backgrounds were used for all experiments. Mice between the ages of 10 weeks and 6 months were used for behavior tests. Animals were randomly assigned to experimental groups with balanced sex representation. Only naive adult animals were used for behavior tests and each animal received only one treatment except for the paw luminance assay where a cross-over approach was used: the mice received either saline or sumatriptan at 15 days post-SNI surgery for the paw luminance test, and two weeks later the mice received the opposite treatment at 29 days post-surgery. All SNI mice were tested by dynamic brush at 7 days post-surgery to confirm the development of allodynia. SNI mice with an allodynia score of 1.5 or higher were used for subsequent tests within at least a one-week interval, whereas mice with a score lower than 1.5 were excluded. Otherwise, all mice including outliers were included in the analyses.

The *Gpr19^{fl}* mouse line was generated in this study at the Gene Targeting and Transgenics Facility at Janelia Research Campus using standard homologous recombination techniques in hybrid mouse embryonic stem (ES) cells. The only coding exon (exon 5) of *Gpr19* was flanked by *loxP* sites. Chimera mice were generated by blastocyst injection. Germline transmission was confirmed by genotyping and the NeoR/KanR selection cassette was removed by crossing to a germline Flp line, *Actb^{FlpE}* [91].

The HEK293T cell line used for *in vitro* signaling assay was not formally authenticated or tested for mycoplasma contamination. However, cell health and identity were monitored routinely throughout the study by visual inspection of cell morphology and assessment of proliferation rate. No morphological abnormalities or unexpected changes in growth rate were observed.

The L5 human DRG tissue was purchased from AnaBios. The tissue was acutely collected from one postmortem human donor without a history of neurological disease, and freshly frozen using dry-ice cooled isopentane. Upon arrival, the tissue was embedded in Neg-50 and stored at −80°C.

METHOD DETAILS

Drug treatments

Tamoxifen solution was prepared as previously described. To generate *Htr1b* or *Gpr19* conditional knockout mice using *Avil^{CreER}* or *Calca^{CreER}*, 1 mg of tamoxifen was delivered daily by intraperitoneal injection for 5 consecutive days from postnatal day 28. For calcium imaging of wildtype mice using *Calca^{CreER}*, 1 mg of tamoxifen was delivered intraperitoneally one week before dissection for DRGs.

Sumatriptan succinate (Sigma) was dissolved in saline and intraperitoneally (i.p.) injected 30 min before behavior assays at 300 μg/kg for most of the behavior tests or 30 mg/kg for high-dose experiments unless otherwise specified.

Zymosan (Sigma) was dissolved in saline at 5 mg/mL. 20 μL of zymosan solution was injected subcutaneously into the plantar surface of the left hind paw.

Gabapentin was dissolved in saline and i.p. injected 30 min before behavioral test at 30 mg/kg.

In situ hybridization (RNAscope)

The standard protocol from ACDBio website was used with a few modifications. Mouse DRGs were freshly dissected, embedded in Neg-50 and flash frozen. Freshly frozen mouse and human DRGs were cryosectioned at 20 μm thickness. Slides were fixed in pre-chilled 4% PFA at 4°C for 1 h, and then dehydrated in 50%, 70% and 100% ethanol sequentially. Next the sections were treated with RNAscope Hydrogen Peroxide for 10 min and then digested using Protease III for 25 min. RNAscope probes were added onto the slide and incubated at 40°C for 2 h, followed amplification and HRP development according to the protocol. The slides were then mounted using DAPI Fluoromount-G (Southern Biotech). Imaging was done using a confocal microscope (Zeiss LSM 900), and ImageJ was used for quantification.

In vitro cAMP signaling assay

The GloSensor cAMP assay (Promega) was used to assess the cAMP level in live cells. HEK293T cells were cultured using standard protocol. 1.5×10^4 cells were seeded per well in tissue culture-treated 96-well white plates with clear flat bottom. Plates were incubated in a 37°C incubator overnight. Cells were then transfected with 50–80 ng of the pGloSensor-22F cAMP plasmid and the plasmid over-expressing human GPCRs of interest each using 500 ng of polyethylenimine per well. Cells were incubated for 24 h. On the day of experiment, the cells were pre-equilibrated in the equilibration medium (100 μL per well, containing 88 μL CO_2-independent media, 10 μL fetal bovine serum and 2 μL GloSensor cAMP Reagent) for 2 h in room temperature. In a plate-reader (FlexStation3), baseline luminescence was acquired before adding compounds. Cells were pre-treated with varying concentrations of agonists by adding 1 μL of 100× compound stock solution per well with 3 replicate wells. After 5 min incubation, 1 μL of 100× isoproterenol stock solution (10 μM) was added to all wells. Luminescence was measured at 5 min–20 min post-isoproterenol addition. For ligand-independent constitutive activity assessment, baseline luminescence was acquired before compound treatment, and luminescence measurements were taken at multiple time points from 5 to 20 min after isoproterenol addition with 6 replicate wells. Each experiment was repeated at least twice.

DRG neuron dissociation and culture

One week after tamoxifen treatment or two weeks from the first tamoxifen treatment for the *Htr1b* conditional knockout mice, animals were sacrificed and DRGs were immediately dissected in cold HBSS buffer. DRGs were collected in 1 mL of digestion solution (HBSS buffer containing 5 mg/mL dispase, 2 mg/mL collagenase and 0.1 mg/mL DNase I) on ice, and then digested by incubating at 37°C under constant rotation (60 rpm) for 45 min. Digestants were spun down at 200 rcf for 5 min and washed twice with HBSS. Cells were resuspended in 1 mL of DMEM medium (Gibco) containing 1/100 Penicillin-Streptomycin and 1/10 fetal bovine serum, and triturated using fire polished glass pipettes from large to small diameters sequentially. 100 μL of dissociated cell suspension was plated onto each 12 mm glass coverslip that had been treated with 50 μg/mL poly D-lysine and 1 μg/mL laminin overnight in a 24-well plate. Cells were left at room temperature for 30 min to attach to the coverslip. 500 μL of plating medium (DMEM medium containing 1/100 Penicillin-Streptomycin, 1/10 fetal bovine serum, 100 μg/mL NGF, 100 μg/mL BDNF, 10 μg/mL GDNF) was added to each well. The plate was then kept in a 37°C incubator with 10% CO_2.

In vitro calcium imaging

The *in vitro* calcium imaging protocol was adapted from a previous report.[42] After culturing for 48 h, plating medium was removed from the 24-well plate and fresh DMEM medium was added. After 20 min in room temperature, the medium was removed and observation solution (145 mM NaCl, 5 mM KCl, 2 mM $CaCl_2$, 1 mM $MgCl_2$, 10 mM HEPES pH7.0, 1/1000 Penicillin-Streptomycin) was added to the well. The coverslip was then transferred to a customized recording chamber (Warner Instrument) that was situated on an upright fluorescence microscope (Nikon) and connected to a gravity fed perfusion system with a six-channel perfusion valve controller and a pump. Cells were constantly perfused with observation solution.

The perfusion valve controller was connected to and automatically controlled by an Arduino board. DRG neurons were depolarized with 25 mM KCl applied as 15 s pulses with 5 min inter-stimulus intervals. The first two KCl pulses were used to determine the baseline excitability of the neurons. After the second pulse of KCl, agonists or putative agonists of candidate GPCRs were applied for 5 min before a third KCl pulse, which was then followed by a washout period that included two more pulses of KCl (Figure 1E, top). Imaging was done using a camera (Thorlabs) connected to the fluorescence scope. Images were analyzed and quantified using ImageJ. Cell excitability was defined as the maximum $\Delta F/F$ value during each trial. The first two KCl trials were used to determine the baseline excitability before drug treatment. Only cells with comparable excitability for the first two trials were included in the analysis (i.e., difference of excitability <15%). Cells whose excitability of the third trial (drug-treated trial) is 15% lower or higher than the baseline (mean of the first two trials) were considered inhibited or enhanced by the drug.

In vivo calcium imaging

The *in vivo* calcium imaging experiments were performed as previously described.[18] Mice were anesthetized with 2% inhalational isoflurane, with body temperature maintained at 37°C throughout the experiment. The back hair near the lumbar region was removed by shaving and applying Nair. After skin was cleaned and disinfected, an incision was made on top of the lumbar vertebrae, and the paravertebral muscles on L3-L5 were removed. A spinal clamp was used to stabilize the spine. The bone on top of the L4 DRG was removed with a rongeur to expose the DRG. The surgical preparation was then transferred to the platform under an upright epifluorescence microscope (Zeiss) with a 10× objective. A 470 nm LED (Thorlabs) was used as the light source. A CMOS Camera (Thorlabs) was triggered at 10 fps with 50 ms exposure time. All stimuli were synchronized with the camera and LED using a DAQ board (National Instrument). A Peltier device (13 × 12 × 2.5 mm, TE technology) was used to deliver thermal stimulation. Thermal paste (Thermal Grizzly) was evenly applied on the Peltier surface. The Peltier was then gently pressed on the glabrous skin of the hind paw to form a firm contact without applying excessive pressure. A thermocouple microprobe (Physitemp) was inserted between the Peltier and skin as a separate measurement of applied temperature. After 20 s of baseline at 32°C, temperature dropped to 0°C at the rate of 5°C s^{-1}. The temperature was maintained at 0°C for 23.6 s before raising back to the baseline. Pinch stimuli were applied at the end of the trial to confirm the viability of cells. Only cells that responded to pinch were included in the analyses.

ImageJ plugin "moco"[92] and "Unsharp mask" filter were used for motion correction and spatial high-pass filtering. Individual cells were manually circled and aligned across videos for pinch and thermal stimulation. The calcium signal measurements from ImageJ were analyzed using custom MATLAB codes. Heatmap plots were generated with ΔF/F of individual cells during thermal stimulation period.

Spared nerve injury (SNI)

The surgery was done as previously described.[49] Mice were anesthetized using isoflurane. Eye lubricant was applied to both eyes. The hair on the left thigh was removed using Nair. The exposed skin was disinfected by 70% ethanol and then betadine. An incision was made above the knee joint. Muscles were carefully separated to expose the sciatic nerve bundle. The branching point where the sural nerve was separated from the tibial and the common peroneal was located. The two nerves were tied using non-absorbable silk suture (Henry Schein) down the branching point without touching the sural. A second knot was tied slightly lower from the first one with the absorbable vicryl suture (Covetrus), and a transection was made between the two knots. The muscles were pulled together and the skin was stitched by making 3–4 double knots with the absorbable vicryl suture. Liquid bandage was applied to the skin. The mice were placed in the recovering cage above a heating pad and put back to the home cage after they awoke. The mice were monitored for 5 days post-surgery. Allodynia in SNI mice were validated using the dynamic brush assay 7 days post-surgery. Animals with allodynia scores under 1.5 were excluded from subsequent tests.

Open field

The open field assay was done as previously described.[93] Two days prior to the experiment, mice were moved to the testing room to acclimate to the room and the testing chamber (25 × 25 × 18 cm, length × width × height). On the day of test, mice were singly placed in the chamber and recorded for 5 min using a top-positioned camera at 30 fps. A custom MATLAB code was used to track distance traveled and time spent exploring in the center.

Cold and hot plate

The cold and hot plate assays were done as previously described.[67] Animals were habituated for 20 min in a chamber (14 × 14 × 30 cm) with a metal plate floor set at 26°C for 2 days. On the day of the experiment, mice received a single dose of either vehicle or sumatriptan (30–300 μg/kg) through intraperitoneal injection. 30 min after the injection, mice were singly placed in a chamber with a clear acrylic front side. The floor metal plate was set at 0°C for cold plate or 51°C for hot plate. Mice were kept in the chamber for 1 min for cold plate or 30 s for hot plate. Behaviors were recorded using a front-positioned high-speed camera at 120 fps. Number of nocifensive behaviors (clenching, flicking, guarding or licking of fore paws and hind paws, jumping) and the latency to the first behavior were manually scored by an experimenter blinded to genotypes or treatments.

von Frey (VF)

The von Frey assay was done as previously described.[67] Mice were habituated for 30 min on an elevated metal grid in a clear acrylic chamber for 2 days. Punctate stimulations were delivered to the pads (for non-surgery mice) or the lateral side (for sham and SNI surgery mice) of the left hind paw using a series of filaments (0.008–4 g). For each filament, 5 stimuli were delivered, followed by another 5 after a 30 s interval. Sudden withdraw, guarding, flicking or licking of the paw indicated a positive response. The number of responses were recorded. The lowest force that elicited 5 or more responses out of 10 was considered the 50% threshold.

Dynamic brush

Dynamic brush was done as previously described.[94] Mice were habituated for 30 min on a metal grid in a clear acrylic chamber for 2 days. Three gentle brushes were delivered to the lateral part of the left hind paw using a paintbrush. Brushing continued with 5–10 s intervals for 3 min per trial. 3 trials were performed for each animal. The responses were graded with a score from 0 to 3: 0 for no response, 1 for sustained lifting or withdraw of the ipsilateral hind paw, 2 for lateral kicking, and 3 for liking. The maximal response

for each trial was considered the score for that trial, and the scores for all three trials were averaged. The number of behaviors were also recorded. Animals with a score of 1.5 and higher were considered allodynic.

Pinprick

The pinprick assay was done as previously described.[20] Mice were habituated in VF chambers for 2 days before the experiment. A custom-made pinprick apparatus with a sharp pin (FST) was used. Upward movement of the pin was triggered with a manual push-button. An accelerometer (SparkFun) was attached to the wire grid floor and a circuit was set up to measure conductance between the pin and the mouse paw that rested on a conductive metal grid. A DAQ board (National Instruments) was used to synchronously record the accelerometer signals, the button presses and skin contact signals. Data were collected for 3 min during which an experimenter delivered multiple pinprick stimuli to the plantar surface of the hind paws. Animal behavior was also recorded with a bottom-viewed camera at 200 fps.

Custom MATLAB code was used for data analyses. Each push of a button was considered a trial. Only trials where the pin made strong contact with the paw skin (conductance value for pin-to-skin contact value exceeding 1.5 V) after the button push were included in the data analyses. Stimulus onset was defined as first contact of pin to skin. Movement onset was empirically defined as when the accelerometer value reached 0.15 V. Movement magnitude was computed as area under the curve for accelerometer values across 0.4 s after stimulus onset.

Inflammatory pain (zymosan model)

Inflammatory pain was induced by intraplantar injection of zymosan.[95] Zymosan solution (100 μg in 20 μL) or vehicle (saline) was subcutaneously injected into the left hind paw. Mice then received i.p. injection of saline or sumatriptan 3.5 h post zymosan/vehicle intraplantar injection. Behavior tests were performed 30 min afterward. VF assay was used to test mechanical pain.

The Hargreaves assay was done for thermal hyperalgesia as previously described.[96] Animals were habituated to the Hargreaves apparatus (Ugo Basile) consisting of a glass floor with Plexiglas enclosures for 5 consecutive days (1–2 h per day). Baseline withdrawal latencies were measured prior to treatment, after which mice were assigned to four groups using stratified allocation to ensure comparable baseline distributions and balanced sex representation. On the day of testing, mice received intraplantar injections of either saline or zymosan. At 3.5 h post-injection, sumatriptan or saline was i.p. administered. Thermal withdrawal latency was assessed 30 min post i.p. injection (i.e., approximately 4 h post intraplantar injection). A focused radiant heat source (50% infrared intensity) was applied to the plantar surface of the ipsilateral hind paw, and latency to withdrawal was recorded. The mean of five trials was used for latency. All behavioral testing was performed by an experimenter blinded to treatment conditions.

Temperature preference

The temperature preference assay was done as previously described.[93] Animals were habituated for 20 min on a metal plate floor set at 26°C for 2 days. Two metal plates (14 × 14 × 18 cm) were placed adjacent to each other. The temperature of each plate was controlled separately. A divider was placed in the middle of the chamber. Both plates were set at 30°C to test for place preference. For the cold temperature preference, one plate was set at 30°C (neutral side) and the other at 18°C (cool side). Mice were placed in the chamber and free to explore. The movement was recorded by a top camera at 30 fps and tracked using a custom MATLAB code. The time the mice spent on each side was quantified, and the preference of the cool side was plotted.

Guarding behavior

Naive mice were habituated in the same way as the cold/hot plate test. Guarding behaviors were assessed in SNI mice at 2–3 weeks post-surgery. 30 min post i.p. injection, mice were placed in a chamber with the metal plate floor set at 26°C. Behaviors were recorded for 5 min with a front high-speed camera at 120 fps and manually scored by an experimenter blinded to treatments. Lifting and then prolonged holding of the ipsilateral hind paw was considered guarding behavior. Care was taken to differentiate a normal step during walking from a guarding behavior. All manual scoring was done by the same blinded experimenter using the same criteria throughout the experiments.

Paw luminance assay

The paw luminance assay was done as previously described.[52] Mice were first habituated individually for 30 min in a black recording chamber (see below), followed by a 30-min video recording session (pre-injury session), one day prior to undergoing the SNI procedure. One week after injury, mechanical allodynia was confirmed in all mice using von Frey testing. At two weeks post-injury, mice were randomly assigned to receive either vehicle or drug treatment, with balanced sex distribution across groups. Mice in the drug group received a single intraperitoneal injection of sumatriptan (300 μg/kg), while control mice received an equivalent volume of saline. Following treatment, mice were placed in the recording chamber and recorded for 30 min. Two weeks later, the treatments were crossed over between groups (i.e., vehicle-treated mice received sumatriptan and vice versa), followed by the same recording procedures.

The recording setup and analysis pipeline were identical to those previously described.[52] Briefly, mice were placed in a black acrylic enclosure (18 × 18 × 15 cm) that was enclosed on all sides except the bottom. The enclosure was positioned on a 25 × 25 × 0.5 cm glass platform. Two independent near-infrared (NIR) LED strips (850 nm; Huake Light Electronics) were mounted

10 cm beneath the glass floor and powered by a 12 V DC supply. Video recordings were acquired in darkness using an NIR-sensitive camera (Basler) positioned 30 cm below the glass surface. Video acquisition was performed using Pylon Viewer software (Basler) under standardized camera settings. All recordings were conducted at 25 frames per second with an initial resolution of 1000 $\times$ 1000 pixels, which was subsequently downscaled to 500 $\times$ 500 pixels for high-throughput analysis. Paw surface contact was quantified based on luminance signals from both hind paws, and the luminance ratio was calculated as the average luminance of the injured (ipsilateral) hind paw relative to the uninjured (contralateral) hind paw over the entire 30-min recording session (i.e., 45,000 frames).

Texture preference (sandpaper)

The texture preference assay was done as previously described.[93] Mice were habituated in the chamber for 2 days. Two types of sandpapers (3M) with smooth (400-grit, blue) or rough texture (100-grit, purple) were used. The chamber was divided into two compartments using a divider. Each compartment had one of the two sandpapers or construction papers with matching colors on the floor. Mice were first run in the chamber with color-matching construction papers (blue on one side and purple on the other) on the floor for 10 min (non-texture control, NT). Then the construction papers were replaced with sandpapers of the matching color. Mice were then run in the chamber for 10 min on the textured floors. Video recording was done using a top camera at 30 fps. Tracking and analysis was done using MATLAB.

Novel object test

Mice were habituated in a chamber for 2 days before the experiment. On the day of experiment, mice were allowed to explore in the empty chamber for 5 min and put back to the home cage. A novel object (a wooden block in a novel shape) was introduced to the chamber. The mice were placed back into the chamber with the novel object and stayed for 5 min. Video recording was done using a top camera at 30 fps. The amount of time the mice spent exploring the object was manually scored by an experimenter blinded to the genotype or the treatment.

Balance beam

The balance beam assay was done as previously described.[93] The balance beam rod was constructed of 1 m of matte white acrylic with a flat 12 mm-wide surface. The beam rod was mounted 50 cm above the bench top. A black matte acrylic box was placed at each end of the beam to provide a start and end enclosure for the animal. A soft pad was stretched below the beam to serve as a soft-landing surface to cushion falls. The day prior to the test, animals were room habituated and trained to cross the beam freely. Each animal was placed in the center of the 12 mm-wide rod and encouraged to walk toward the end enclosure containing home cage bedding. The animals were encouraged to walk across the beam at least 3 crossings before returning to the home cage. Once trained, each animal was tested for 4 consecutive crossings with a 5 s rest between each crossing. Both top and side cameras were used to record the crossings. Time to cross and number of slips were manually scored in a blinded manner.

Electrocardiogram

Heart rate was measured using ECGenie Clinic (Mouse Specifics). Mice were habituated on the electrode tower for 30 min. After vehicle or drug administration, mice were immediately placed on the electrode tower. Recording started 5 min post injection, and multiple readings were taken at different time points from 5 to 30 min post injection. Data was analyzed using the EzCG Signal Analyses software (Mouse Specifics).

Slice electrophysiology

P15-P21 acute spinal cord slices were used for whole-cell patch clamp recordings of lamina II dorsal horn neurons as previously described.[97] Mice were briefly anesthetized with isoflurane, and intracardially perfused with ice-cold oxygenated choline solution before spinal cord removal. The isolated spinal cord was embedded in low-melting agarose (Sigma Aldrich), and 300 μm transverse slices were prepared from lumbar levels using a Leica vibrating blade microtome (Leica). Spinal cord slices were prepared in ice-cold oxygenated choline solution containing: 92 mM choline chloride, 2.5 mM KCl, 1.2 mM NaH_2PO_4, 30 mM $NaHCO_3$, 20 mM HEPES, 2.5 mM glucose, 5 mM sodium ascorbate, 2 mM thiourea, 3 mM sodium pyruvate, 10 mM $MgSO_4$, 0.5 mM $CaCl_2$. Slices recovered at 34°C for 30 min in HEPES holding solution equilibrated with 95% O_2, 5% CO_2, containing: 86 mM NaCl, 2.5 mM KCl, 1.2 mM NaH_2PO_4, 35 mM $NaHCO_3$, 20 mM HEPES, 25 mM glucose, 5 mM sodium ascorbate, 2 mM thiourea, 3 mM sodium pyruvate, 1 mM $MgSO_4$, 2 mM $CaCl_2$ (pH 7.3, osmolarity 305).[97] Spinal cord slices were then transferred to a submerged recording chamber at room temperature and continuously perfused with artificial cerebrospinal fluid (aCSF) containing: 2.5 mM $CaCl_2$, 1 mM NaH_2PO_4, 119 mM NaCl, 2.5 mM KCl, 1.3 mM MgSO4, 26 mM $NaHCO_3$, 25 mM dextrose, and 1.3 mM sodium ascorbate, saturated with 95% O_2, 5% CO_2 at a flow rate of ~1–2 mL/min. Patch pipettes (5–7 MΩ) were pulled from borosilicate glass (Sutter Instruments) and filled with an internal solution containing: 135 mM $CsMeSO_3$, 4 mM ATP-Mg^{2+}, 0.3 mM GTP-Na^+, 1 mM EGTA, 3.3 mM QX-314(Cl^- salt), 8 mM Na_2-phoshocreatine and 10 mM HEPES. Neurons were visualized using infrared differential interference contrast and fluorescence microscopy. Whole cell voltage-clamp recordings were obtained from lamina I-II_o dorsal horn neurons under visual guidance using a 40× water-immersion objective. Neurons were voltage-clamped at −70 mV, and input resistance and series resistance were monitored throughout each experiment. Cells were excluded from analysis if these values changed by >20% during the recording.

Excitatory postsynaptic currents (EPSCs) were evoked optogenetically by stimulating $Na_v1.8^{ChR2}$ primary afferent axonal terminals with 1 ms pulses of blue light (473 nm, 5 mW) delivered at 30 s intervals using LED whole field illumination through the water-immersion 40× objective. After a 10 min stable baseline, sumatriptan (10 μM) was bath-applied for 10 min. In a subset of experiments, optically evoked EPSCs (oEPSCs) were blocked by NBQX (10 μM) confirming that they were mediated by AMPARs. Data were acquired using a Multiclamp 700B amplifier, a Digidata 1440A acquisition system, and Clampfit software (Molecular Devices). Sampling rate was 10 kHz, and data were low-pass filtered at 3 kHz. No correction for junction potential was applied. oEPSC peak amplitudes were measured, and responses were normalized to the mean of the 10 min baseline period. Normalized values were then used for average response plots, with all cells time-aligned at the beginning of the 10-min baseline. Averaged oEPSC amplitudes for the 2 min period immediately before sumatriptan application were compared with averaged EPSC amplitudes during the 2 min period at 8–10 min after sumatriptan bath-application (just before wash-out).

QUANTIFICATION AND STATISTICAL ANALYSIS

Data analysis and quantification were performed by manual scoring in a blinded fashion or automatic tracking using MATLAB; all custom code is publicly available at Zenodo (https://doi.org/10.5281/zenodo.20801945) or described previously.[98] GraphPad Prism 10 was used for statistical analyses. All data are shown as the mean ± standard error of the mean (SEM). Comparisons between two groups were done by a two-tailed Student's *t* test and significance was assigned at $p < 0.05$ (* for $p < 0.05$, ** for $p < 0.01$).

Cell Reports Medicine, Volume *7*

Supplemental information

Targeting $G_{i/o}$-coupled GPCRs to inhibit nociceptors: Insights from the serotonin receptor Htr1b and triptans

Jing Peng, Brianna T. Sanchez, Anda M. Chirila, Xiangsunze Zeng, Michelle M. DeLisle, Lijun Qi, Jia Yin Xiao, Karina Lezgiyeva, Sarah A. Low, Clifford J. Woolf, Nikhil Sharma, and David D. Ginty

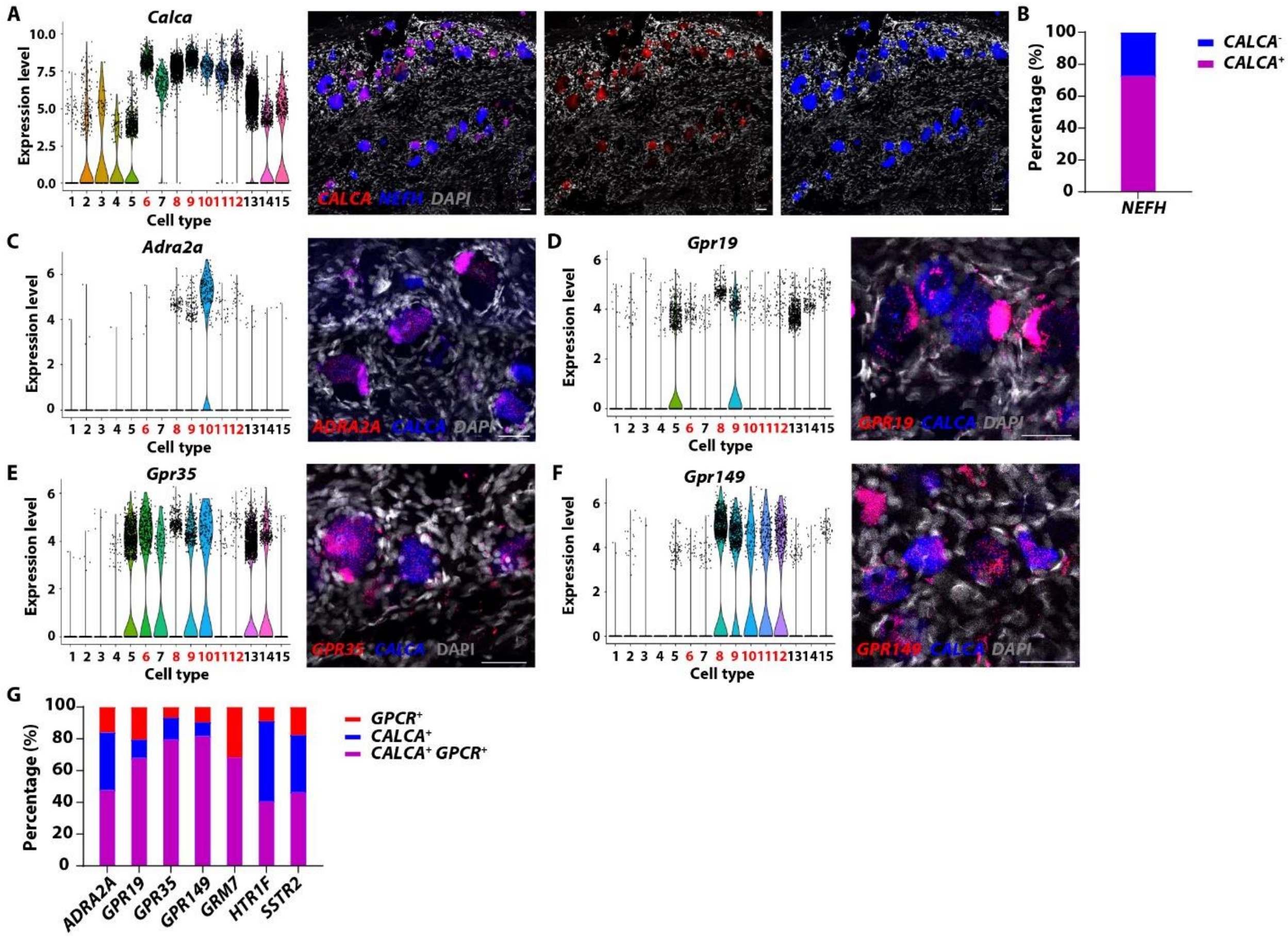

Figure S1. Expression of a selected subset of GPCR candidates validated by RNAscope in human DRGs, related to Figure 1. (**A**) Expression of *CALCA* in human DRGs was assessed by RNAscope (right), with the mouse *Calca* expression across all DRG neuron subtypes in our RNA-seq dataset shown for comparison (left). For all the violin plots in this figure, the cell types are number coded with the $CGRP^+$ subtypes highlighted in red: 1. Proprioceptors; 2. Aβ-SA LTMRs; 3. Aβ-RA LTMRs; 4. Aδ-LTMRs; 5. C-LTMRs; 6. C-HTMR/Heat (*Mrgpra3*$^+$); 7. C-HTMR/Heat (*Mrgprb4*$^+$); 8. C-Heat (*Sstr2*$^+$); 9. CGRP-ε (*Oprk1*$^+$); 10. CGRP-γ (*Adra2a*$^+$); 11. Aδ-HTMRs (*Bmpr1b*$^+$); 12. Aδ-HTMR/Heat (*Smr2*$^+$); 13. C-HTMR/Heat (*Mrgprd*$^+$); 14. C-HTMR/Heat (*Sst*$^+$); 15. C-Cold (*Trpm8*$^+$). Scale bar: 50 µm. (**B**) Quantifications of (A). (**C-F**) A subset of GPCRs were co-stained with *CALCA* in human DRGs by RNAscope: *ADRA2A* (C), *GPR19* (D), *GPR35* (E), *GPR149* (F). Scale bar: 50 µm. (**G**) Quantification of the co-expression of GPCRs with *CALCA* in human DRGs.

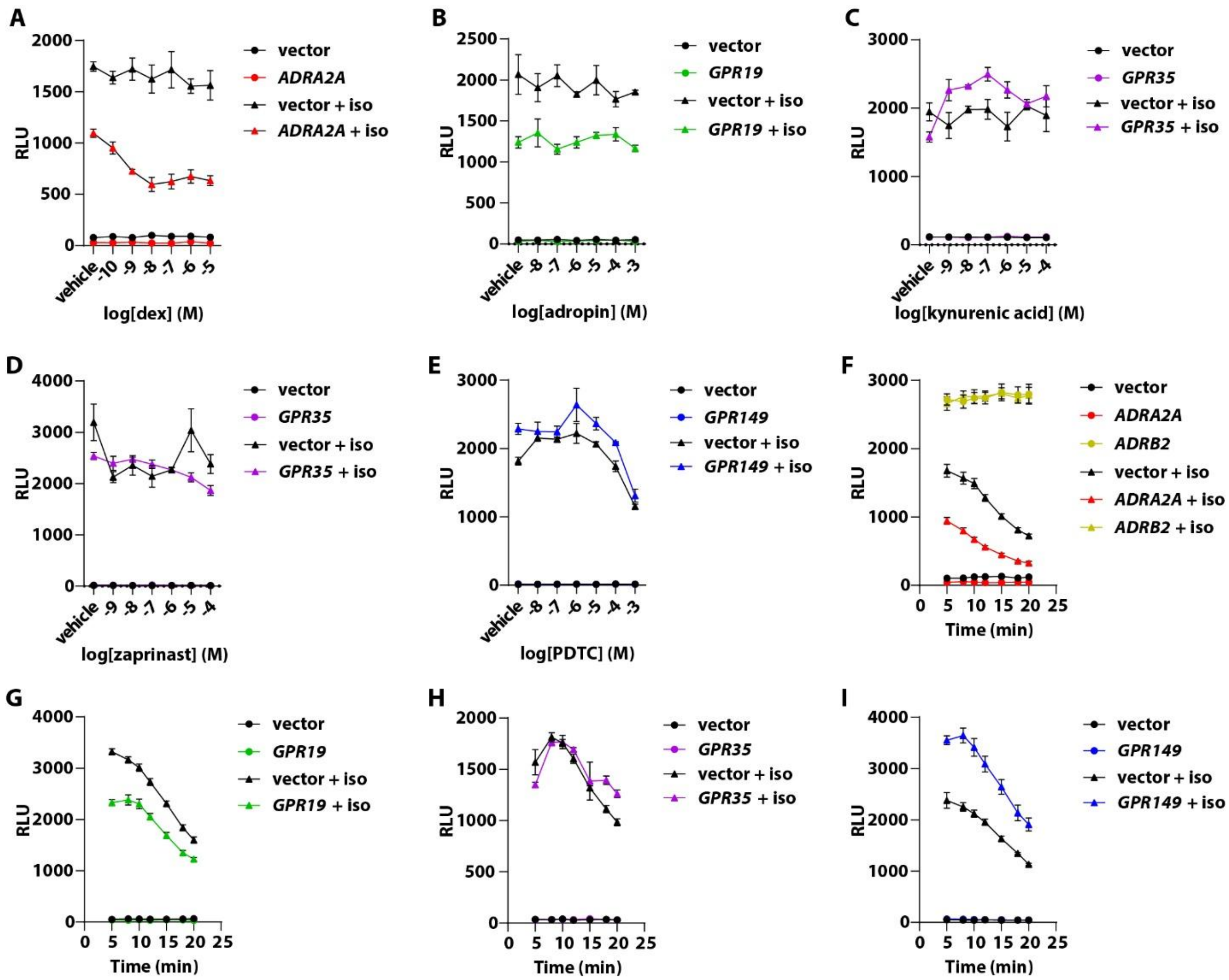

Figure S2. Select orphan GPCRs were tested for $G_{i/o}$-coupling activities using an *in vitro* cAMP signaling assay, related to Figure 1. (**A**) Dexmedetomidine dose-dependently inhibited cAMP accumulation in *ADRA2A* over-expressing cells. cAMP signals are shown as relative luminescence unit (RLU). (**B**) Adropin did not affect cAMP levels in *GPR19* over-expressing cells. (**C, D**) Neither kynurenic acid (C) nor zaprinast (D) affected cAMP levels in *GPR35* over-expressing cells. (**E**) PDTC reduced cAMP accumulation at higher doses in both *GPR149* over-expressing and vector-expressing cells. (**F**) Compared to the vector-expressing control, cells over-expressing *ADRB2* had a significantly higher cAMP level, which was not further increased by its agonist isoproterenol (iso), while cells over-expressing *ADRA2A* have a lower cAMP level, consistent with their G_s- and $G_{i/o}$-coupling activity respectively. (**G**) Cells over-expressing *GPR19* exhibited a diminished level of cAMP, suggesting constitutive $G_{i/o}$-coupling activity. (**H**) Over-expression of *GPR35* did not affect cAMP levels. (**I**) Over-expressing *GPR149* increased cAMP levels, suggesting constitutive G_s-coupling activity. Data in this and all other supplemental figures are represented as mean +/- SEM.

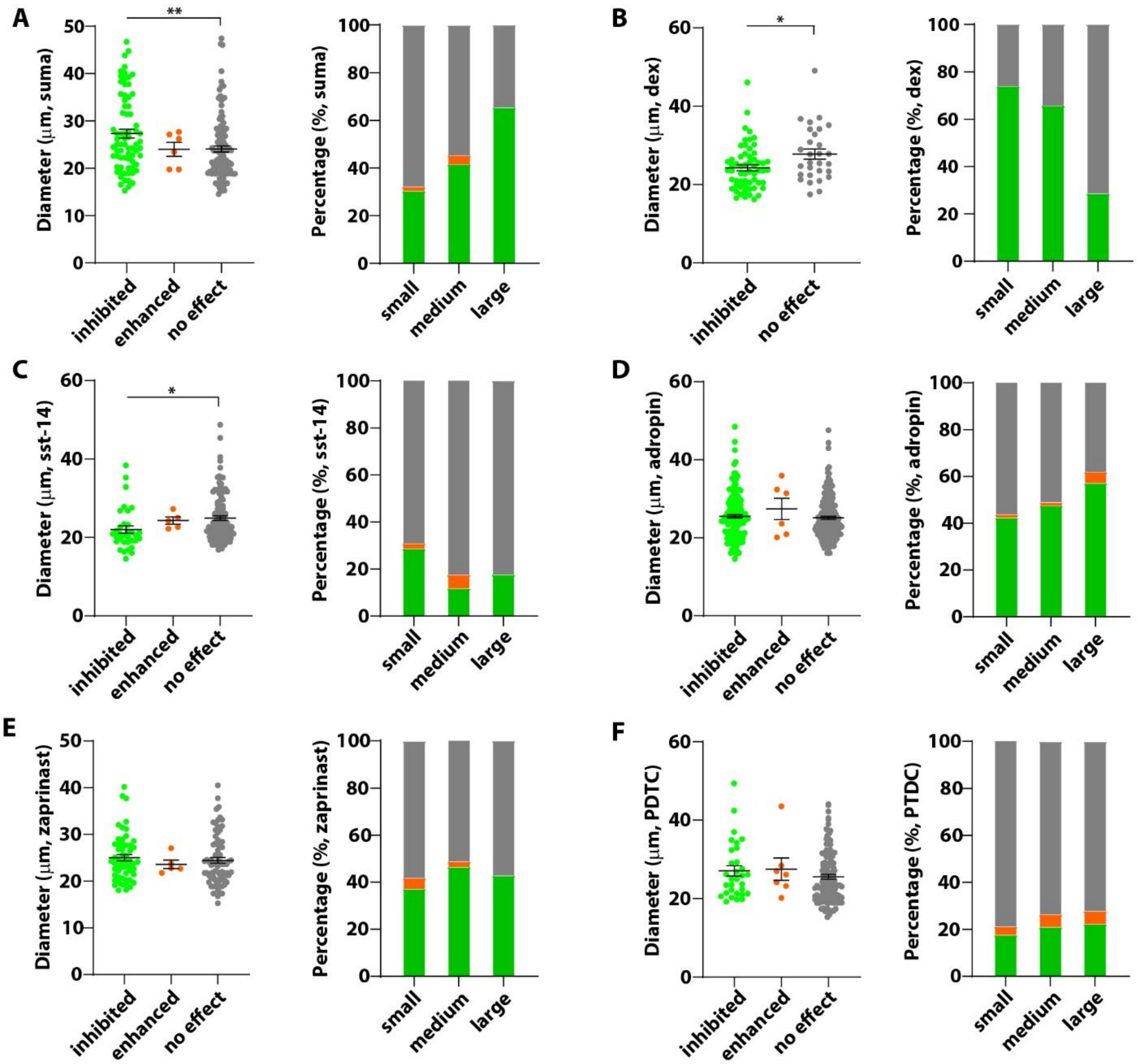

Figure S3. Cell size distribution of the *in vitro* calcium imaging assay, related to Figure 1. (**A**) Cells that are inhibited by sumatriptan have larger diameters than the non-responding cells (left). Each dot represents a cell. A higher percentage of large diameter cells are inhibited by sumatriptan than small- or medium-diameter cells (right). (**B, C**) Dexmedetomidine (dex, **B**) and sst-14 (**C**) preferentially inhibit small diameter cells, consistent with the expression of the α2-adrenergic receptor and the somatostatin receptor in small diameter DRG neurons. Cells treated with adropin (**D**), zaprinast (**E**) or PDTC (**F**) had a relatively uniform size distribution, indicating likely more than one targets. Green: inhibited. Orange: increased. Gray: no effect. All statistical comparisons were performed using a two-tailed Student's t-test. *$p < 0.05$, **$p < 0.01$.

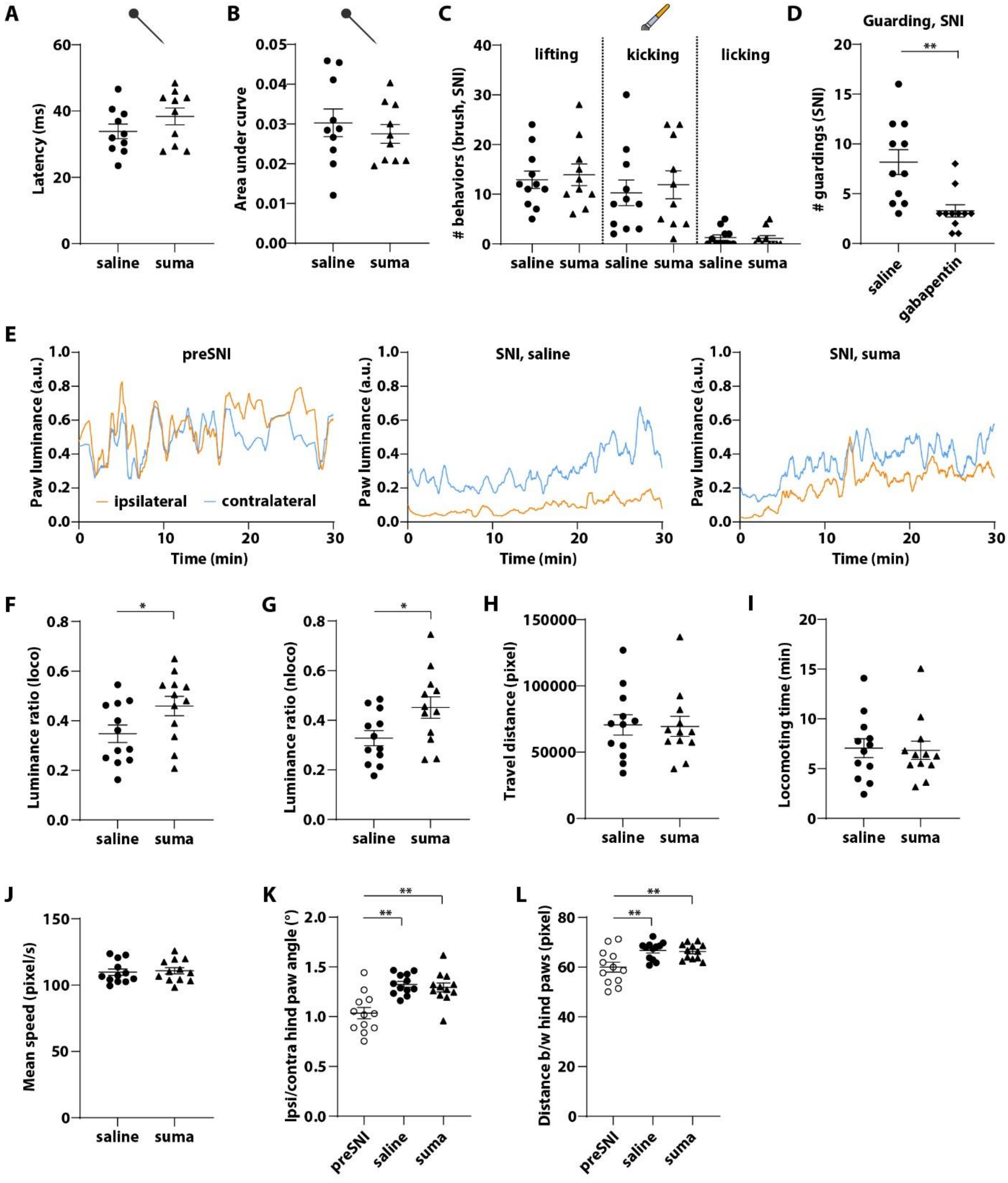

Figure S4. Sumatriptan did not affect acute mechanical pain, dynamic allodynia, locomotion or gait of the SNI mice, related to Figure 2. (**A, B**) Sumatriptan had no effect on acute mechanical pain in the pinprick assay. Latency (A) and cumulative response magnitude (area under the curve of response magnitude between 0-0.4 s) (B) were not different between the treatment groups. (**C**) Sumatriptan did not reduce the number of nocifensive behaviors in the dynamic brush test (**D**) Gabapentin (30 mg/kg, i.p.) significantly reduced guarding behaviors in SNI

mice. (**E**) Representative traces of paw luminance of ipsilateral (orange) and contralateral hind paw (blue) before SNI (left), after SNI treated with saline (middle) or sumatriptan (right). The paw luminance values were scaled (normalized by the maximum value) and smoothed before plotting, and shown in arbitrary unit (a.u.). The SNI animals had significantly reduced paw luminance of the ipsilateral paw compared to the contralateral hind paw. Sumatriptan partially alleviated the decrease of luminance of the ipsilateral hind paw. (**F, G**) Sumatriptan increased the paw luminance ratio of the SNI mice during both the locomoting (F) and non-locomoting phase (G). (**H-J**) Sumatriptan did not affect travel distance (H), locomoting time (I) or speed (J) of the SNI mice. (**K, L**) Sumatriptan did not affect the gait of the SNI mice, including the ipsilateral hind paw angle (normalized to contralateral hind paw) (K) and the distance between the hind paws (L).

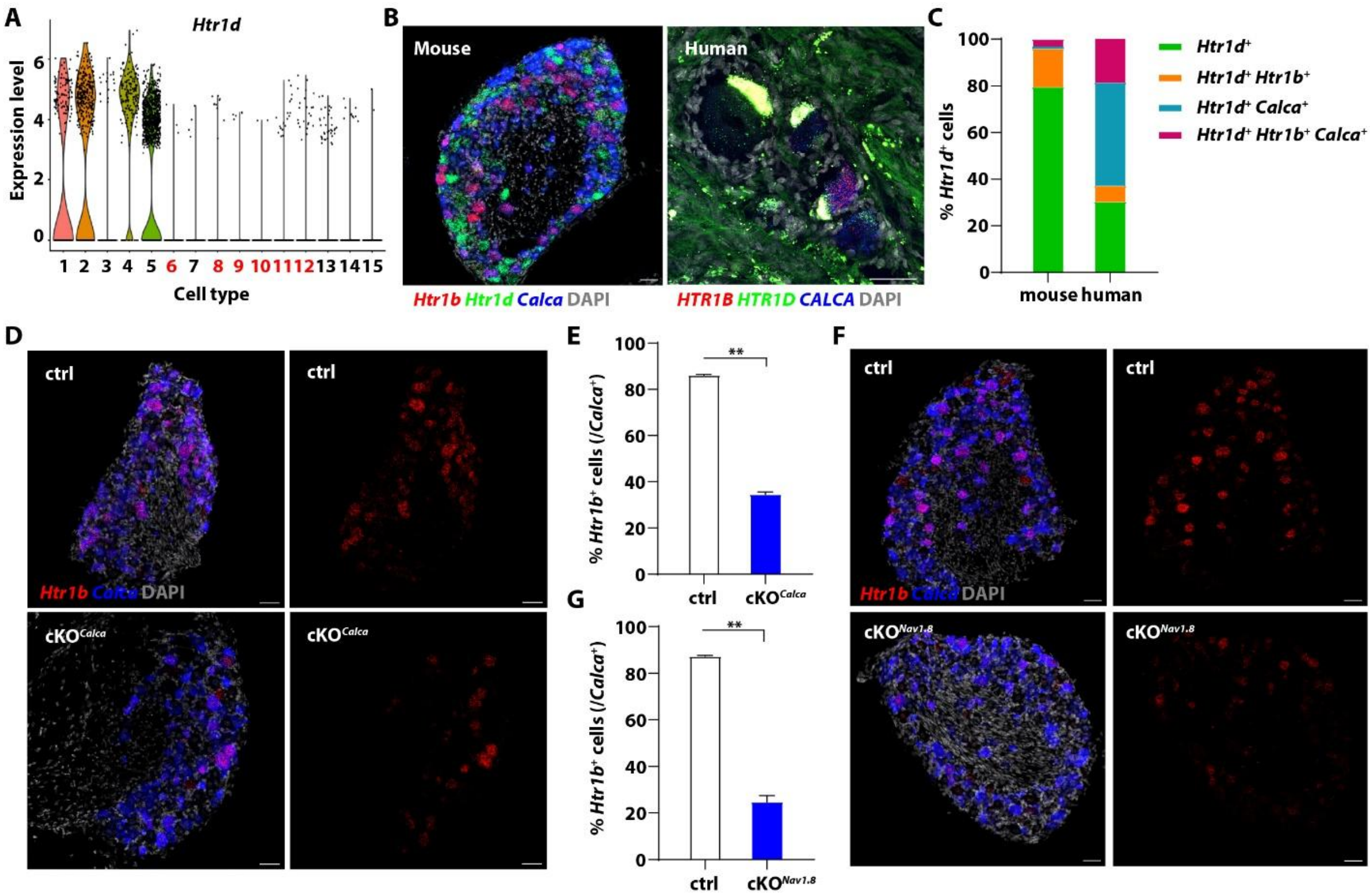

Figure S5. Htr1b was efficiently depleted in the cKO*Calca* mice used for the *in vitro* calcium imaging and slice electrophysiology experiments, related to Figure 3. (**A**) Expression of *Htr1d* in our mouse DRG single cell RNA-seq dataset. The cell types are number coded with the CGRP$^+$ subtypes highlighted in red: 1. Proprioceptors; 2. Aβ-SA LTMRs; 3. Aβ-RA LTMRs; 4. Aδ-LTMRs; 5. C-LTMRs; 6. C-HTMR/Heat (*Mrgpra3*$^+$); 7. C-HTMR/Heat (*Mrgprb4*$^+$); 8. C-Heat (*Sstr2*$^+$); 9. CGRP-ε (*Oprk1*$^+$); 10. CGRP-γ (*Adra2a*$^+$); 11. Aδ-HTMRs (*Bmpr1b*$^+$); 12. Aδ-HTMR/Heat (*Smr2*$^+$); 13. C-HTMR/Heat (*Mrgprd*$^+$); 14. C-HTMR/Heat (*Sst*$^+$); 15. C-Cold (*Trpm8*$^+$). (**B**) Expression of *Htr1b*, *Htr1d* and *Calca* was assessed by RNAscope. *Htr1d* expression did not overlap with *Htr1b* or *Calca* in mouse (left) and human DRGs (right). The arrow indicates a cell expressing *HTR1D* but not *HTR1B* or *CALCA* in the human DRG (right). Scale bar: 50 μm. (**C**) Quantification of (B, N=3 for mouse DRG and N=1 for human DRG). (**D, E**) RNAscope assay confirmed efficient depletion of *Htr1b* in the cKO*Calca* mice (N=3 for each genotype). Scale bar: 50 μm. (**F**) RNAscope showed significantly reduced *Htr1b* signal in the DRGs of the cKO*Nav1.8* mice. Scale bar: 50 μm. (**G**) Quantification of (F, N=3 for each genotype).

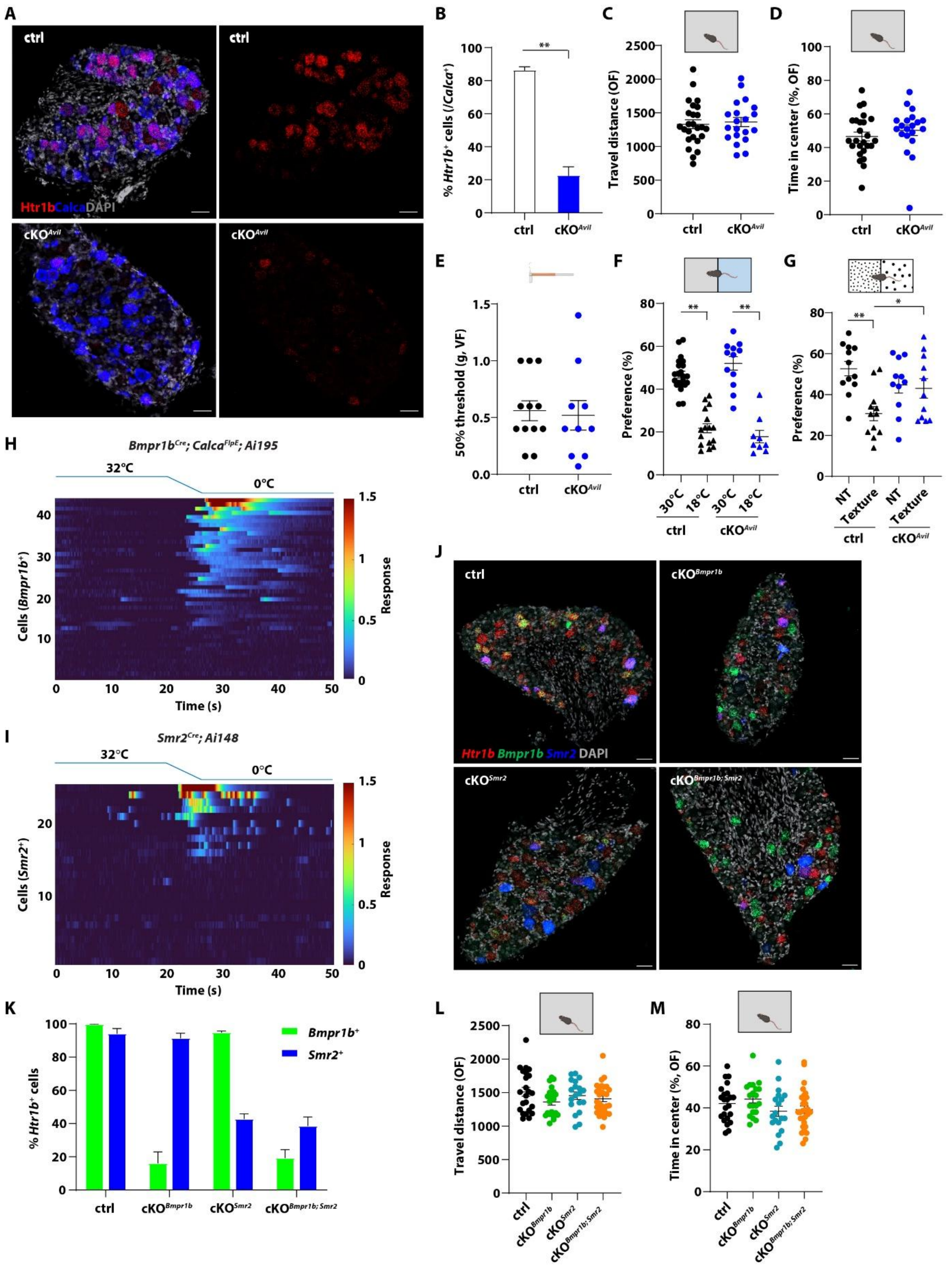

Figure S6. Characterization of the pan-sensory neuron and subtype-specific *Htr1b* cKO mice, related to Figure 4. (**A, B**) RNAscope confirmed efficient depletion of *Htr1b* in the cKO^{Avil} mice (A), quantified in (B, N=3

for both control and cKO^{Avil}). Scale bar: 50 µm. (**C, D**) The *Htr1b* cKO^{Avil} mice behaved normally in the open field (OF) test, exhibiting comparable travel distance (C) and time in center (D) to the control littermates. (**E, F**) The *Htr1b* cKO^{Avil} mice showed normal mechanical and thermal sensitivity as tested by von Frey assay (E) and temperature preference assay (F). (**G**) In the sandpaper test, *Htr1b* cKO^{Avil} mice exhibited no preference for the smooth or rough texture, in contrast to control littermates which demonstrated strong aversion to the rough texture. NT: non-texture, construction paper on both sides. Texture: sandpaper 400 vs 100 grit. Preference is defined as the percentage of time the animal spent on the rough side, i.e. 100 grit sandpaper (Texture), or the same side but with construction paper (NT). (**H, I**) A subset of $Bmpr1b^{+}$ (E, N=2, n=44) and $Smr2^{+}$ (F, N=3, n=25) DRG neurons responded to noxious cold (0°C) in the *in vivo* calcium imaging assay. Thermal stimuli were delivered to the glabrous skin of the mouse hind paw using a Peltier, while the ipsilateral L4 DRG was imaged. The thermal stimuli started with physiological skin surface temperature (32°C) as the baseline, and then ramped (5°C s^{-1}) and held (20 s) at 0°C. Each row of the heatmap represents responses of an individual neuron. (**J, K**) RNAscope confirmed efficient deletion of *Htr1b* in the cKO^{Bmpr1b}, cKO^{Smr2} or $cKO^{Bmpr1b; Smr2}$ mice (J), with quantification shown in (K, N=3 for each genotype). Scale bar: 50 µm. (**L, M**) The subtype-specific *Htr1b* cKO mice performed normally in the open field test, showing comparable travel distance (L) and time in center (M) to the control littermates.

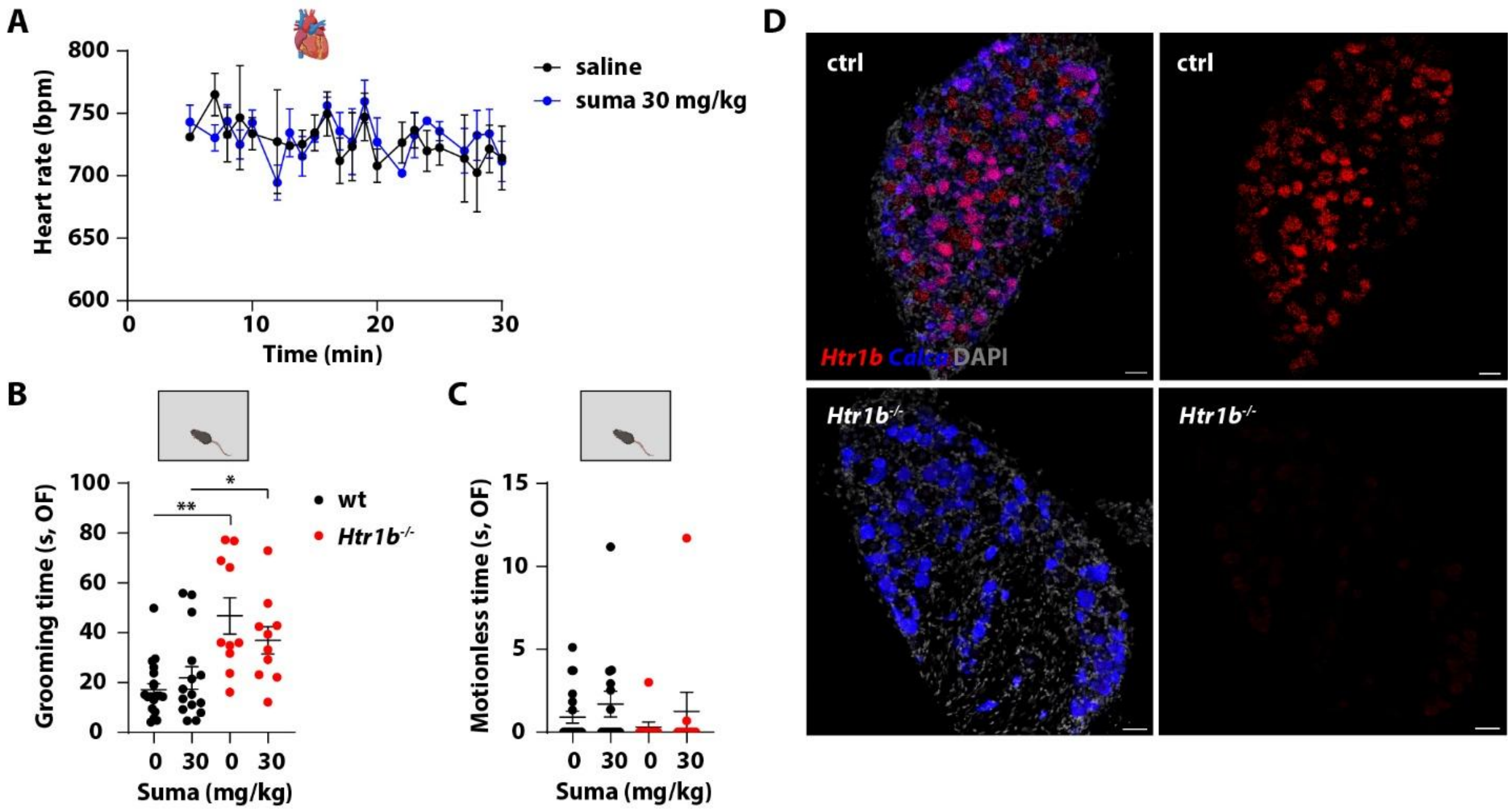

Figure S7. A high dose of sumatriptan didn't affect cardiac function or cause sedation-like behavior, related to Figure 5. (**A**) Mice receiving a high dose of sumatriptan (30 mg/kg) showed normal heart rate compared to saline-treated animals (N=10 for both groups). (**B**) The high dose of sumatriptan did not affect grooming behavior, although *Htr1b*$^{-/-}$ mice (red) exhibited enhanced grooming compared to the control mice (black). (**C**) The high dose of sumatriptan did not cause a significant increase of motionless time in both control (black) and *Htr1b*$^{-/-}$ mice (red). (**D**) RNAscope confirmed complete loss of *Htr1b* signal in DRGs of *Htr1b*$^{-/-}$ mice (N=3). Scale bar: 50 μm.

Gene	Family	Expression pattern in mouse DRG	G protein coupling	Expression in human DRG
Adgrf5	Adhesion Class GPCRs	C-HTMR/Heat (*Mrgpra3*$^+$, *Mrgprd*$^+$)	$G_{q/11}$	–
Adora2b	Adenosine receptors	C-HTMR/Heat (*Mrgpra3*$^+$, *Mrgprd*$^+$)	G_s, secondary: $G_{q/11}$	–
Adra2a	Adrenoceptors	CGRP-γ (*Adra2a*$^+$), C-Heat (*Sstr2*$^+$), CGRP-ε (*Oprk1*$^+$)	$G_{i/o}$, secondary: G_s	Co-expression with *CALCA*
Agtr1b	Angiotensin receptors	C-HTMR/Heat (*Mrgpra3*$^+$, *Sst*$^+$), CGRP-ε (*Oprk1*$^+$), CGRP-γ (*Adra2a*$^+$)	$G_{i/o}$, $G_{q/11}$	–
Avpr1a	Vasopressin and oxytocin receptors	C-Heat (*Sstr2*$^+$)	$G_{q/11}$	–
Calcrl	Calcitonin receptors	CGRP-ε (*Oprk1*$^+$), C-Heat (*Sstr2*$^+$), CGRP-γ (*Adra2a*$^+$), C-HTMR/Heat (*Sst*$^+$)	–	–
Ccr10	Chemokine receptors	C-Heat (*Sstr2*$^+$), C-Cold (*Trpm8*$^+$)	$G_{i/o}$	–
Crhr2	Corticotropin-releasing factor receptors	C-Heat (*Sstr2*$^+$), C-Cold (*Trpm8*$^+$)	G_s, secondary: $G_{q/11}$	–
Cysltr2	Leukotriene receptors	C-HTMR/Heat (*Sst*$^+$)	$G_{q/11}$, secondary: $G_{i/o}$	–
Drd1	Dopamine receptors	Aδ-HTMRs (*Bmpr1b*$^+$)	G_s	–
F2r	Proteinase-activated receptors	CGRP-γ (*Adra2a*$^+$)	–	–
F2rl1	Proteinase-activated receptors	C-HTMR/Heat (*Sst*$^+$)	–	–
Fzd4	Class Frizzled GPCRs	Aδ-HTMRs (*Bmpr1b*$^+$), CGRP-γ (*Adra2a*$^+$), C-HTMR/Heat (*Mrgpra3*$^+$)	$G_{12/13}$	–
Fzd5	Class Frizzled GPCRs	C-HTMR/Heat (*Mrgpra3*$^+$, *Sst*$^+$, *Mrgprd*$^+$), CGRP-γ (*Adra2a*$^+$), CGRP-ε (*Oprk1*$^+$), C-Heat (*Sstr2*$^+$)	$G_{q/11}$	–
Galr1	Galanin receptors	C-Heat (*Sstr2*$^+$), CGRP-ε (*Oprk1*$^+$), CGRP-γ (*Adra2a*$^+$), C-HTMR/Heat (*Mrgpra3*$^+$)	$G_{i/o}$	No expression
Ghsr	Ghrelin receptor	CGRP-γ (*Adra2a*$^+$)	$G_{q/11}$, secondary: $G_{i/o}$, $G_{12/13}$	–
Gper1	G protein-coupled estrogen receptor	C-HTMR/Heat (*Sst*$^+$)	$G_{i/o}$, secondary: G_s	–
Gpr135	Class A Orphans	C-Heat (*Sstr2*$^+$), CGRP-ε (*Oprk1*$^+$), C-Cold (*Trpm8*$^+$), C-HTMR/Heat (*Sst*$^+$, *Mrgpra3*$^+$), CGRP-γ (*Adra2a*$^+$)	–	–
Gpr149	Class A Orphans	C-Heat (*Sstr2*$^+$), CGRP-ε (*Oprk1*$^+$), CGRP-γ (*Adra2a*$^+$), Aδ-HTMRs (*Bmpr1b*$^+$), Aδ-HTMR/Heat (*Smr2*$^+$)	–	Co-expression with *CALCA*
Gpr150	Class A Orphans	C-HTMR/Heat (*Sst*$^+$, *Mrgpra3*$^+$), CGRP-γ (*Adra2a*$^+$), CGRP-ε (*Oprk1*$^+$), C-Cold (*Trpm8*$^+$)	–	–
Gpr160	Class A Orphans	CGRP-ε (*Oprk1*$^+$), C-Heat (*Sstr2*$^+$), C-HTMR/Heat (*Sst*$^+$, *Mrgprd*$^+$)	–	–
Gpr165[a]	–	C-Heat (*Sstr2*$^+$)	–	–
Gpr174	Class A Orphans	Aδ-HTMRs (*Bmpr1b*$^+$), CGRP-γ (*Adra2a*$^+$), CGRP-ε (*Oprk1*$^+$), C-Cold (*Trpm8*$^+$)	–	–
Gpr179	Class C Orphans	C-HTMR/Heat (*Mrgpra3*$^+$, *Sst*$^+$, *Mrgprd*$^+$), CGRP-ε (*Oprk1*$^+$), CGRP-γ (*Adra2a*$^+$)	–	–

Gpr19	Class A Orphans	C-Heat (*Sstr2*$^+$), C-Cold (*Trpm8*$^+$), CGRP-ε (*Oprk1*$^+$), C-HTMR/Heat (*Sst*$^+$, *Mrgpra3*$^+$), CGRP-γ (*Adra2a*$^+$)	–	Co-expression with *CALCA*
Gpr26	Class A Orphans	C-Heat (*Sstr2*$^+$), C-Cold (*Trpm8*$^+$)	G_s	–
Gpr35	Class A Orphans	C-HTMR/Heat (*Mrgpra3*$^+$, *Sst*$^+$, *Mrgprd*$^+$), CGRP-γ (*Adra2a*$^+$), C-Heat (*Sstr2*$^+$), CGRP-ε (*Oprk1*$^+$), C-LTMRs	–	Co-expression with *CALCA*
Gpr4	Class A Orphans	C-HTMR/Heat (*Sst*$^+$)	G_s, $G_{q/11}$, $G_{12/13}$	–
Gpr45	Class A Orphans	C-Heat (*Sstr2*$^+$), C-HTMR/Heat (*Sst*$^+$, *Mrgpra3*$^+$, *Mrgprd*$^+$), C-Cold (*Trpm8*$^+$), CGRP-ε (*Oprk1*$^+$), CGRP-γ (*Adra2a*$^+$), Aδ-HTMRs (*Bmpr1b*$^+$)	–	No expression
Grm5	Metabotropic glutamate receptors	CGRP-ε (*Oprk1*$^+$), Aδ-HTMRs (*Bmpr1b*$^+$), CGRP-γ (*Adra2a*$^+$), C-HTMR/Heat (*Mrgpra3*$^+$)	$G_{q/11}$, secondary: G_s, $G_{i/o}$	–
Grm7	Metabotropic glutamate receptors	C-Heat (*Sstr2*$^+$), C-Cold (*Trpm8*$^+$), CGRP-γ (*Adra2a*$^+$), CGRP-ε (*Oprk1*$^+$), Aδ-HTMRs (*Bmpr1b*$^+$), C-HTMR/Heat (*Mrgprd*$^+$, *Mrgpra3*$^+$)	$G_{i/o}$	Broad expression
Hcrtr1	Orexin receptors	Aδ-HTMRs (*Bmpr1b*$^+$), C-Heat (*Sstr2*$^+$), CGRP-ε (*Oprk1*$^+$), CGRP-γ (*Adra2a*$^+$)	$G_{q/11}$	–
Hrh1	Histamine receptors	C-HTMR/Heat (*Mrgpra3*$^+$, Sst+), CGRP-γ (*Adra2a*$^+$)	$G_{q/11}$	–
Hrh2	Histamine receptors	C-HTMR/Heat (*Sst*$^+$, *Mrgpra3*$^+$)	$G_{q/11}$, secondary: G_s	–
Htr1a	5-Hydroxytryptamine receptors	C-HTMR/Heat (*Sst*$^+$), CGRP-ε (*Oprk1*$^+$)	$G_{i/o}$	No expression
Htr1b	5-Hydroxytryptamine receptors	C-Heat (*Sstr2*$^+$), Aδ-HTMRs (*Bmpr1b*$^+$), Aδ-HTMR/Heat (*Smr2*$^+$), CGRP-γ (*Adra2a*$^+$), CGRP-ε (*Oprk1*$^+$)	$G_{i/o}$	Co-expression with *CALCA*
Htr1f	5-Hydroxytryptamine receptors	C-HTMR/Heat (*Sst*$^+$)	$G_{i/o}$	Co-expression with *CALCA* but not *SST*
Mas1	Class A Orphans	Aδ-HTMRs (*Bmpr1b*$^+$)	$G_{i/o}$, $G_{q/11}$	–
Mrgpra1[b]	Class A Orphans	C-HTMR/Heat (*Mrgpra3*$^+$), CGRP-ε (*Oprk1*$^+$)	$G_{q/11}$	–
Mrgpra2a[b]	Class A Orphans	C-HTMR/Heat (*Mrgpra3*$^+$, *Mrgprd*$^+$, *Sst*$^+$), CGRP-ε (*Oprk1*$^+$), CGRP-γ (*Adra2a*$^+$)	$G_{q/11}$	–
Mrgpra2b[b]	Class A Orphans	C-HTMR/Heat (*Mrgpra3*$^+$, *Mrgprd*$^+$, *Sst*$^+$), CGRP-ε (*Oprk1*$^+$), CGRP-γ (*Adra2a*$^+$)	$G_{q/11}$	–
Mrgpra3[b]	Class A Orphans	C-HTMR/Heat (*Mrgpra3*$^+$)	$G_{q/11}$	–
Mrgpra4[b]	Class A Orphans	C-HTMR/Heat (*Mrgpra3*$^+$)	$G_{q/11}$	–
Mrgprb5[b]	Class A Orphans	C-HTMR/Heat (*Mrgpra3*$^+$, *Mrgprd*$^+$)	$G_{q/11}$	–
Mrgprx1[b]	Class A Orphans	C-HTMR/Heat (*Mrgpra3*$^+$), CGRP-ε (*Oprk1*$^+$)	$G_{q/11}$	–
Npffr1	Neuropeptide FF/neuropeptide AF receptors	CGRP-γ (*Adra2a*$^+$), C-Heat (*Sstr2*$^+$), Aδ-HTMRs (*Bmpr1b*$^+$), C-Cold (*Trpm8*$^+$)	$G_{i/o}$	–
Npy1r	Neuropeptide Y receptors	C-Heat (*Sstr2*$^+$), CGRP-γ (*Adra2a*$^+$), CGRP-ε (*Oprk1*$^+$)	$G_{i/o}$	–
Npy2r	Neuropeptide Y receptors	C-HTMR/Heat (*Sst*$^+$), Aδ-HTMRs (*Bmpr1b*$^+$), CGRP-γ (*Adra2a*$^+$), CGRP-ε (*Oprk1*$^+$)	$G_{i/o}$, secondary: $G_{q/11}$	No expression

Ntsr2	Neurotensin receptors	C-Heat (*Sstr2*$^+$), C-Cold (*Trpm8*$^+$), CGRP-ε (*Oprk1*$^+$), CGRP-γ (*Adra2a*$^+$)	$G_{q/11}$	–
Opn3	Opsin receptors	C-Heat (*Sstr2*$^+$), CGRP-ε (*Oprk1*$^+$), CGRP-γ (*Adra2a*$^+$)	–	–
Oprk1	Opioid receptors	CGRP-ε (*Oprk1*$^+$)	$G_{i/o}$, secondary: $G_{12/13}$	–
Oprm1	Opioid receptors	C-Heat (*Sstr2*$^+$), C-Cold (*Trpm8*$^+$), C-HTMR/Heat (*Sst*$^+$, *Mrgpra3*$^+$), CGRP-ε (*Oprk1*$^+$), CGRP-γ (*Adra2a*$^+$)	$G_{i/o}$, secondary: $G_{q/11}$	–
P2ry2	P2Y receptors	C-HTMR/Heat (*Mrgpra3*$^+$), Aδ-HTMRs (*Bmpr1b*$^+$), CGRP-γ (*Adra2a*$^+$), C-Heat (*Sstr2*$^+$), Aδ-HTMR/Heat (*Smr2*$^+$)	$G_{q/11}$, secondary: $G_{i/o}$, $G_{12/13}$	–
Prokr2	Prokineticin receptors	Aδ-HTMRs (*Bmpr1b*$^+$), CGRP-γ (*Adra2a*$^+$)	$G_{q/11}$, secondary: G_s, $G_{i/o}$	–
Ptafr	Platelet-activating factor receptor	C-HTMR/Heat (*Sst*$^+$)	$G_{i/o}$, $G_{q/11}$	–
Ptgdr	Prostanoid receptors	C-Heat (*Sstr2*$^+$), C-HTMR/Heat (*Mrgprd*$^+$, *Mrgpra3*$^+$)	G_s	–
Ptger1	Prostanoid receptors	CGRP-ε (*Oprk1*$^+$), C-Heat (*Sstr2*$^+$), CGRP-γ (*Adra2a*$^+$), C-HTMR/Heat (*Sst*$^+$)	$G_{q/11}$, secondary: $G_{i/o}$	–
Ptgir	Prostanoid receptors	CGRP-ε (*Oprk1*$^+$), CGRP-γ (*Adra2a*$^+$), Aδ-HTMRs (*Bmpr1b*$^+$), C-Heat (*Sstr2*$^+$), Aδ-HTMR/Heat (*Smr2*$^+$), C-HTMR/Heat (*Mrgpra3*$^+$, *Sst*$^+$)	G_s, secondary: $G_{i/o}$, $G_{q/11}$	–
Rho	Opsin receptors	C-Heat (*Sstr2*$^+$), CGRP-ε (*Oprk1*$^+$), CGRP-γ (*Adra2a*$^+$), Aδ-HTMRs (*Bmpr1b*$^+$)	–	–
Rxfp3	Relaxin family peptide receptors	CGRP-γ (*Adra2a*$^+$)	$G_{i/o}$	–
S1pr1	Lysophospholipid (S1P) receptors	C-HTMR/Heat (*Sst*$^+$), CGRP-ε (*Oprk1*$^+$), C-Heat (*Sstr2*$^+$), CGRP-γ (*Adra2a*$^+$)	$G_{i/o}$	–
S1pr2	Lysophospholipid (S1P) receptors	C-HTMR/Heat (*Sst*$^+$, *Mrgpra3*$^+$), C-Heat (*Sstr2*$^+$)	G_s, $G_{q/11}$, $G_{12/13}$	–
S1pr3	Lysophospholipid (S1P) receptors	C-Heat (*Sstr2*$^+$), CGRP-ε (*Oprk1*$^+$), CGRP-γ (*Adra2a*$^+$), Aδ-HTMRs (*Bmpr1b*$^+$)	$G_{i/o}$, $G_{q/11}$, $G_{12/13}$	–
Sstr1	Somatostatin receptors	CGRP-γ (*Adra2a*$^+$), CGRP-ε (*Oprk1*$^+$)	$G_{i/o}$	–
Sstr2	Somatostatin receptors	C-Heat (*Sstr2*$^+$)	$G_{i/o}$	Co-expression with *CALCA*
Tbxa2r	Prostanoid receptors	CGRP-γ (*Adra2a*$^+$)	$G_{q/11}$	–
Vmn1r85[c]	Vomeronasal receptors	CGRP-ε (*Oprk1*$^+$), CGRP-γ (*Adra2a*$^+$)	–	–
Vmn1r89[c]	Vomeronasal receptors	CGRP-ε (*Oprk1*$^+$)	–	–
Vmn2r113[c]	Vomeronasal receptors	C-HTMR/Heat (*Mrgpra3*$^+$)	–	–

Table S1. List of GPCRs that are selectively expressed in CGRP$^+$ sensory neurons, related to Figure 1. The GPCRs that are restrictively expressed in one or more CGRP$^+$ DRG sensory neuron subtypes were identified by mapping individual genes to our single cell RNA-seq dataset. Expression patterns are listed. Information on the transduction pathways were obtained from the IUPHAR/BPS Guide to PHARMACOLOGY. Expression patterns in human DRGs inferred from the RNAscope data are also included. [a] Human ortholog *GPR165P* is a pseudogene. [b] Not well conserved between mouse and human. [c] No human ortholog.
